# Red Cell Distribution Width as an Independent Prognostic Biomarker in MASLD

**DOI:** 10.3390/antiox15091065

**Published:** 2026-08-25

**Authors:** Tom Ryu, Jaejun Lee, Ji Won Han, Hyun Yang, Keungmo Yang

**Affiliations:** 1 Department of Internal Medicine, Institute for Digestive Research, Digestive Disease Center, Soonchunhyang University College of Medicine, Seoul 04401, Republic of Korea; tomryu1@schmc.ac.kr; 2 Department of Internal Medicine, Division of Gastroenterology and Hepatology, College of Medicine, The Catholic University of Korea, Seoul 06591, Republic of Korea; pwln0516@catholic.ac.kr (J.L.); tmznjf@catholic.ac.kr (J.W.H.); oneggu@catholic.ac.kr (H.Y.)

**Keywords:** red cell distribution width, metabolic dysfunction-associated steatotic liver disease, UK Biobank, cardiovascular disease, prognostic biomarker

## Abstract

Metabolic dysfunction-associated steatotic liver disease (MASLD) requires practical and inexpensive tools for risk stratification. We investigated whether the red cell distribution width (RDW), a routinely available hematologic parameter, predicts adverse clinical outcomes, using data from 352,623 UK Biobank participants, including 165,700 individuals with MASLD. RDW was analyzed as both a continuous and categorical variable using Cox proportional hazards models, restricted cubic splines, inverse probability treatment weighting, landmark, competing risk, and mediation analyses. Over a median follow-up of 13.6 years, each 1% increase in RDW was independently associated with higher risks of all-cause mortality (hazard ratio [HR] 1.14), cardiovascular disease (HR 1.07), cerebrovascular disease (HR 1.07), and chronic kidney disease (HR 1.09) (all *p* < 0.001). These associations remained consistent across multiple sensitivity analyses, and conventional biomarkers explained a modest proportion of the observed associations. Oxidative stress, together with other systemic processes affecting erythrocyte homeostasis, represents one potential mechanism underlying these associations. Higher RDW was independently associated with adverse clinical outcomes after adjustment for routine liver and metabolic biomarkers, supporting its potential value as a readily available prognostic marker in MASLD.

## 1. Introduction

Metabolic dysfunction-associated steatotic liver disease (MASLD) was introduced to describe steatotic liver disease in the presence of cardiometabolic risk, replacing terminology that depended on exclusionary and potentially stigmatizing language [1,2]. Fatty liver disease affects approximately one-third of adults worldwide and continues to increase with obesity and metabolic dysfunction [3].

That distinction is difficult, because prognosis in MASLD is shaped by several partially overlapping biological axes. Fibrosis stage remains the strongest hepatic predictor of adverse outcomes, but cardiovascular disease, cerebrovascular disease, chronic kidney disease, and mortality often determine the long-term clinical trajectories [4,5]. Therefore, contemporary guidelines emphasize risk stratification using cardiometabolic features, liver enzymes, and noninvasive fibrosis tests such as the Fatty Liver Index (FLI), the non-alcoholic fatty liver disease (NAFLD) fibrosis score (NFS), and the fibrosis-4 (FIB-4) index [6,7]. These tools are useful, but they remain imperfect surrogates for the systemic inflammatory, nutritional, hematologic, and renal perturbations that accompany high-risk MASLD phenotypes.

Red cell distribution width (RDW), a standard component of the complete blood count, quantifies heterogeneity in erythrocyte size. Although RDW was originally used to support the differential diagnosis of anemia, it has since emerged as a prognostic marker across heart failure, coronary disease, chronic kidney disease, population cohorts, and chronic inflammatory states [8,9,10]. Biologically, RDW is not a liver enzyme and is not a direct fibrosis marker. Rather, it integrates ineffective erythropoiesis, inflammation-mediated impairment of erythrocyte maturation, micronutrient and protein calorie stress, and altered red cell survival [11,12]. Experimental and clinical studies have also demonstrated that oxidative stress contributes to erythrocytes’ membrane damage, impaired red blood cell deformability, and anisocytosis, thereby increasing RDW [13,14]. Because oxidative stress contributes to both MASLD’s pathophysiology and alterations in erythrocyte homeostasis, it represents one potential biological mechanism linking higher RDW with adverse outcomes in MASLD.

Several liver-disease studies have linked RDW or RDW-derived indices to liver fibrosis and disease severity [15,16,17]. For instance, a Korean cohort reported that elevated RDW was associated with advanced fibrosis in ultrasonography-defined NAFLD, and biopsy-based hepatitis B cohorts found that RDW, platelet count, albumin, and the RDW-to-platelet ratio were related to fibrosis or necroinflammation [15,18]. These studies provided important preliminary evidence but were cross-sectional or disease-specific, had limited longitudinal follow-up, and could not determine whether RDW predicted hard clinical endpoints in the modern MASLD spectrum.

Therefore, we used the UK Biobank to address three pre-specified objectives in eligible participants, including a MASLD cohort, followed for a median of 13.6 years. We examined whether baseline RDW independently predicts all-cause mortality, cardiovascular disease, cerebrovascular disease, and chronic kidney disease across the SLD spectrum; whether these associations are consistent across multiple analytical approaches, including inverse probability treatment weighting (IPTW), landmark analysis, competing risk modelling, and sensitivity analyses for unmeasured confounding; and how much of the observed associations can be accounted for by 10 conventional clinical biomarkers spanning liver injury, hepatic synthesis, and hematologic, glycemic, lipid, and renal pathways.

## 2. Materials and Methods

### 2.1. Study Design and Participants

The UK Biobank is a prospective cohort of approximately 502,000 adults recruited between 2006 and 2010 in the United Kingdom. Baseline assessment included questionnaires, physical measurements, blood sampling, and linkage to hospital and death registry records [19]. Written informed consent was provided by all participants, and the present work used de-identified data under an approved UK Biobank application. The UK Biobank Application ID for this research is 117214, with ethical approval obtained from the North West Multi-Centre Research Ethics Committee.

**Figure 1:**
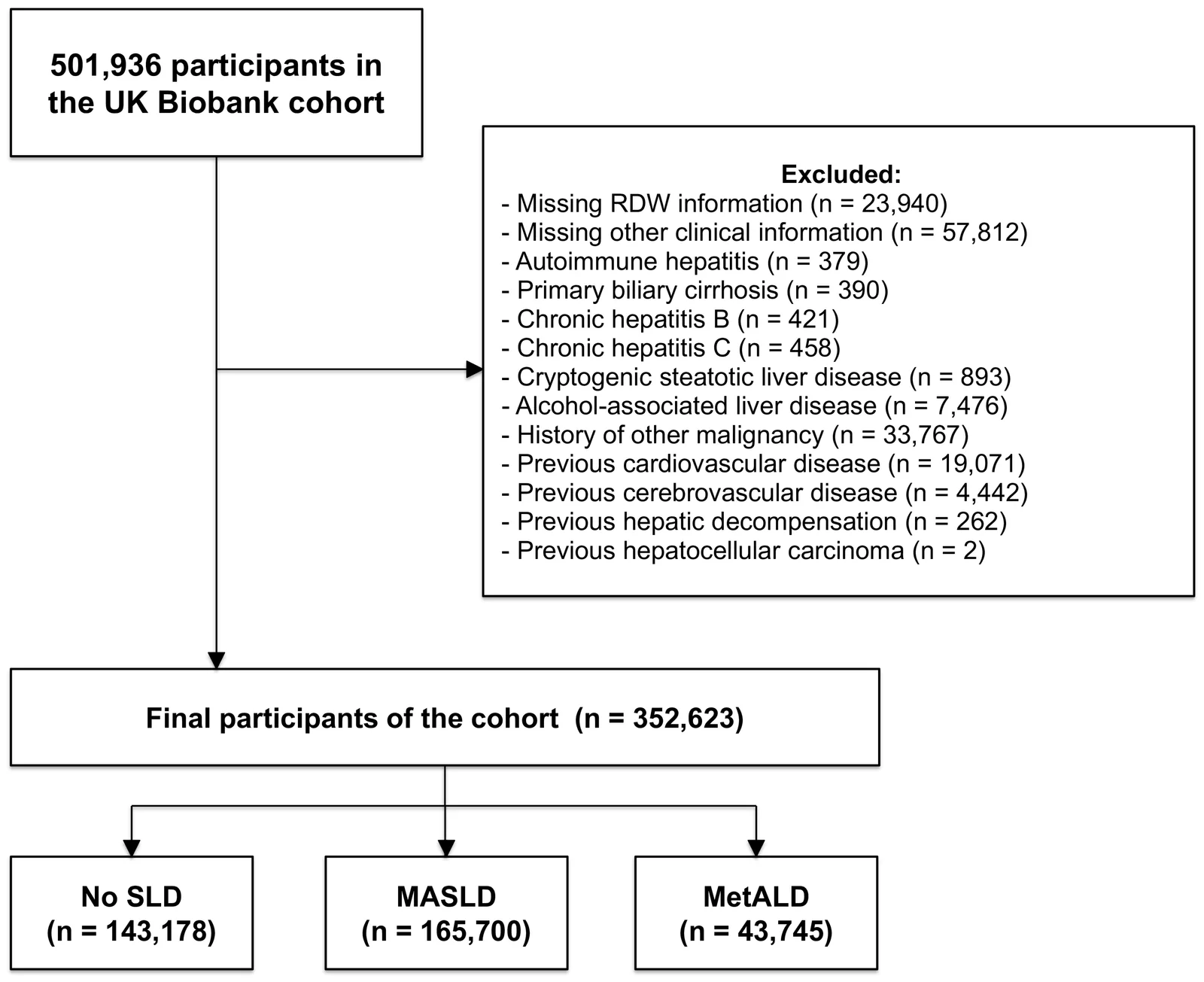
Study flowchart and analytic cohort derivation. Flow diagram showing participants’ selection, exclusion criteria, and final classification into the No SLD, MASLD, and MetALD cohorts. RDW, red cell distribution width; SLD, steatotic liver disease; MASLD, metabolic dysfunction-associated steatotic liver disease; MetALD, MASLD with increased alcohol intake.

Participants were excluded for missing RDW, chronic viral hepatitis, autoimmune hepatitis, primary biliary cholangitis, cryptogenic steatotic liver disease, alcohol-associated liver disease, prevalent malignancy, previous cardiovascular disease, previous cerebrovascular disease, previous hepatic decompensation, or previous hepatocellular carcinoma. For each non-mortality endpoint, participants with the corresponding event before baseline were also excluded from the relevant outcome analysis. The final analytic cohort comprised 352,623 participants: 143,178 with no steatotic liver disease (No SLD), 165,700 with MASLD, and 43,745 with MASLD with increased alcohol intake (MetALD) (Figure 1).

### 2.2. Definitions of Steatotic Liver Disease Cohorts

Hepatic steatosis was diagnosed using the Fatty Liver Index (FLI), which combines body mass index (BMI), waist circumference, triglycerides, and gamma-glutamyl transferase (GGT); an FLI ≥ 60 was used to define hepatic steatosis [20]. Accordingly, MASLD in the present study represents an operational epidemiological definition based on FLI-defined steatosis rather than an imaging- or histology-confirmed diagnosis. Participants were assigned to the mutually exclusive No SLD, MASLD, and MetALD cohorts according to their steatosis status, cardiometabolic risk features, and alcohol intake, consistent with the 2023 nomenclature framework. MASLD was defined as the presence of hepatic steatosis together with at least one cardiometabolic risk factor, including (i) body mass index ≥ 25 kg/m^2^ (≥23 kg/m^2^ in Asians) or increased waist circumference; (ii) fasting glucose ≥ 100 mg/dL, HbA1c ≥ 5.7%, or Type 2 diabetes; (iii) blood pressure ≥ 130/85 mmHg or antihypertensive treatment; (iv) triglycerides ≥ 150 mg/dL or lipid-lowering therapy; or (v) high density lipoprotein (HDL) cholesterol ≤ 40 mg/dL in men or ≤50 mg/dL in women. MetALD was defined in individuals meeting the MASLD criteria who reported alcohol consumption of 140–350 g/week in women or 210–420 g/week in men [1]. FIB-4 and the NFS were used as noninvasive fibrosis stratifiers, not as study outcomes. The FIB-4 categories were low (<1.30), indeterminate (1.30–2.67), and high (≥2.67). The NFS categories were low (<−1.455), indeterminate (−1.455 to <0.676), and high (≥0.676) [21,22].

### 2.3. Exposure, Outcomes, and Covariates

The primary exposure was RDW measured at baseline (UK Biobank field 30070), and serial RDW measurements during follow-up were not incorporated into the analyses. RDW was modelled per 1% absolute increase. RDW was additionally analyzed per one standard deviation for marker comparison analyses and by cohort-specific quartile for Kaplan–Meier, cumulative incidence, and IPTW analyses. RDW quartiles were derived within the analytic cohort and labelled as Q1 (lowest) through Q4 (highest). Q1, representing the lowest RDW levels, was selected as the reference group to facilitate interpretation of the graded association with progressively higher RDW levels. RDW quartiles were defined according to the baseline RDW distribution as Q1 (≤12.90%), Q2 (>12.90 to ≤13.34%), Q3 (>13.34 to ≤13.87%), and Q4 (>13.87%). Four defined endpoints were analyzed: all-cause mortality, cardiovascular disease, cerebrovascular disease, and chronic kidney disease. Mortality was identified by national death register linkage. Incident disease endpoints were identified by linked hospital and registry records using predefined International Classification of Diseases (10th Revision) (ICD-10) code groupings, including I20–I25 for cardiovascular disease, I60–I69 for cerebrovascular disease, and N18 for chronic kidney disease. Follow-up was measured from baseline assessment to the first event, death, censoring, or the latest available linked record date. Covariates were assembled in nested models. Model 1 was unadjusted. Model 2 was adjusted for age and sex. Model 3 was further adjusted for body mass index, diabetes mellitus, hypertension, dyslipidemia, and smoking status. Model 4, the primary fully adjusted model, further included aspartate aminotransferase (AST), alanine aminotransferase (ALT), GGT, platelet count, albumin, and creatinine.

### 2.4. Statistical Analysis

Continuous variables are summarized as the mean ± standard deviation or median (interquartile range), and categorical variables as the count (percentage). Baseline characteristics were compared across RDW quartiles using one-way analysis of variance or chi-square tests, as appropriate. Cox proportional hazards models estimated the association between RDW and each endpoint. The proportional hazards assumption was evaluated using Schoenfeld residuals. Although statistical evidence of non-proportionality was observed for some models, inspection of the scaled Schoenfeld residuals did not indicate substantial time-dependent patterns. Restricted cubic splines with four knots at the 5th, 35th, 65th, and 95th percentiles evaluated the dose–response relationships. Wald tests assessed overall association, and likelihood-ratio tests assessed nonlinearity. Kaplan–Meier methods were used to characterize time-to-event patterns. For non-fatal outcomes, the primary Cox models were interpreted as cause-specific hazard models, while competing-risk analyses accounting for death were performed as sensitivity analyses. For non-fatal outcomes, the primary Cox models were interpreted as cause-specific hazard models, while competing risk analyses accounting for death were performed as sensitivity analyses. To address measured confounding, stabilized multinomial IPTW was estimated for RDW quartile assignment, conditional on Model 4’s covariates and trimmed at the 1st and 99th percentiles, and balance was assessed using maximum absolute standardized mean differences across pairwise quartile contrasts. Reverse causation was examined by a two-year landmark analysis. Subgroup analyses were intended to descriptively assess the consistency of the associations across clinically significant subgroups rather than to formally test for effect modification.

Exploratory associational mediation analysis was performed to assess the extent to which candidate biomarkers statistically accounted for the RDW–outcome association in the MASLD cohort using the difference method on the log-hazard scale. Ten candidate mediators were considered: AST, ALT, GGT, total bilirubin, albumin, platelet count, glucose, triglyceride, HDL cholesterol, and creatinine. Mediators were analyzed individually, by biological pathway, and jointly. Hazard ratios (HRs) with 95% confidence intervals (CIs) were estimated using Cox proportional hazards models. The proportion mediated was calculated as (log HRtotal − log HRdirect)/log HRtotal × 100. Bootstrap 95% CIs used 500 resamples. Negative mediated proportions were reported as observed, consistent with suppressor behavior rather than truncated. Analyses were conducted in R version 4.5.0. Two-sided *p* values < 0.05 were considered statistically significant.

## 3. Results

### 3.1. Baseline Characteristics

The final analytic cohort included 352,623 participants, of whom 165,700 met the operational definition of MASLD. In the MASLD cohort, the mean age was 56.2 ± 8.0 years, 96,166 participants (58.0%) were male, the mean BMI was 29.6 ± 4.3 kg/m^2^, and the mean RDW was 13.5 ± 0.9%. Across RDW quartiles, Q4 participants were older than Q1 participants (57.1 vs. 54.9 years), had higher BMI (30.5 vs. 29.0 kg/m^2^), were more often current smokers (12.4% vs. 7.7%), and had higher prevalences of diabetes (16.3% vs. 11.4%), hypertension (51.7% vs. 44.1%), and dyslipidemia (33.0% vs. 30.2%). Sex distribution shifted with increasing RDW, with fewer men in Q4 than Q1 (51.1% vs. 59.8%). Compared with Q1, Q4 had significantly lower ALT (25.1 vs. 28.0 U/L), GGT (41.3 vs. 43.4 U/L), and albumin (4.5 vs. 4.6 g/dL), while platelet count was significantly higher (263.7 vs. 250.3 × 10^9^/L) (Table 1). Similar baseline gradients were present in the total, No SLD, and MetALD cohorts (Supplementary Tables S1–S3).

### 3.2. RDW and Clinical Outcomes Across Cohorts

**Figure 2:**
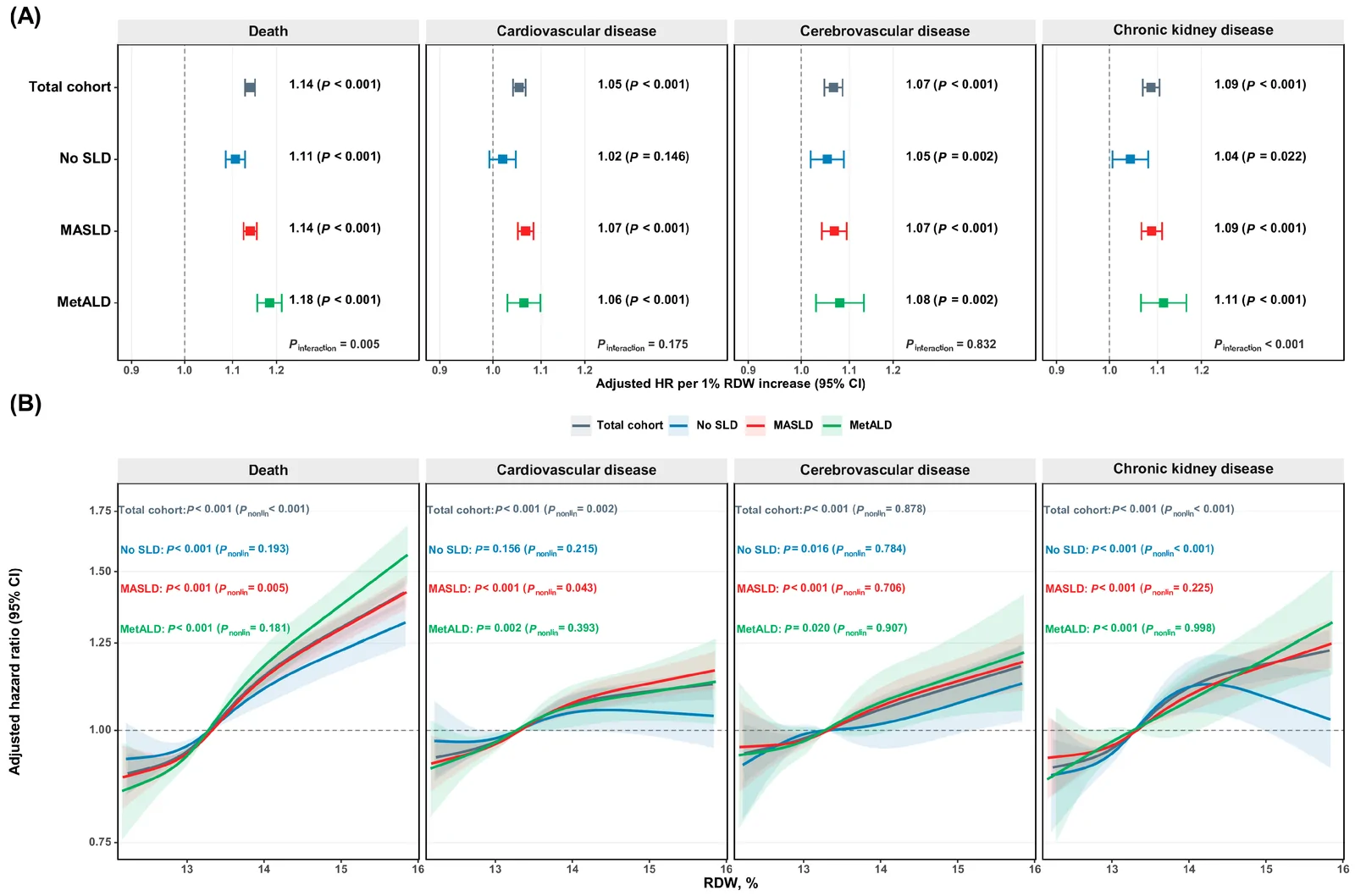
Associations of RDW with clinical outcomes across the SLD spectrum. (**A**) Forest plots of fully adjusted HRs per 1% increase in RDW for all-cause mortality, cardiovascular disease, cerebrovascular disease, and chronic kidney disease in the total, No SLD, MASLD, and MetALD cohorts. (**B**) Restricted cubic spline curves showing dose–response associations between RDW and clinical outcomes across cohorts. Solid lines indicate adjusted HRs, and shaded bands indicate 95% CIs. The vertical dashed line indicates an HR of 1.0 (null effect). The horizontal dashed line indicates an HR of 1.0 (reference). Overall and nonlinearity *p* values are shown in each panel. RDW, red cell distribution width; SLD, steatotic liver disease; MASLD, metabolic dysfunction-associated steatotic liver disease; MetALD, MASLD with increased alcohol intake; HR, hazard ratio; CI, confidence interval.

In the MASLD cohort, each 1% absolute increase in RDW was independently associated with all four endpoints under fully adjusted Model 4: death (HR 1.14, *p* < 0.001), cardiovascular disease (HR 1.07, *p* < 0.001), cerebrovascular disease (HR 1.07, *p* < 0.001), and chronic kidney disease (HR 1.09, *p* < 0.001) (Figure 2A). The same direction of association was observed in the total, No SLD, and MetALD cohorts. Quartile-based Cox models in MASLD showed a graded relationship. In Model 4, Q4 versus Q1 was associated with higher hazards of death (HR 1.45, 95% CI 1.38–1.52), cardiovascular disease (HR 1.21, 95% CI 1.16–1.26), cerebrovascular disease (HR 1.23, 95% CI 1.15–1.32), and chronic kidney disease (HR 1.42, 95% CI 1.34–1.52), with a *p*-trend < 0.001 for every endpoint (Table 2). The same direction of association was observed in the total, No SLD, and MetALD cohorts, with comparable effect sizes (Supplementary Tables S4–S6). Restricted cubic splines showed monotone dose–response patterns across the observed RDW range. In the MASLD cohort, overall associations were statistically significant for all endpoints (all *p* overall < 0.001). Evidence of nonlinearity was modest for death (*p* nonlinearity = 0.005) and cardiovascular disease (*p* nonlinearity = 0.043), whereas nonlinearity was not observed for cerebrovascular disease (*p* nonlinearity = 0.706) or chronic kidney disease (*p* nonlinearity = 0.225). Overall, the predominant pattern was a continuous increase in risk with higher RDW levels (Figure 2B).

### 3.3. Quartile-Resolved Event Curves and Subgroup Analyses

**Figure 3:**
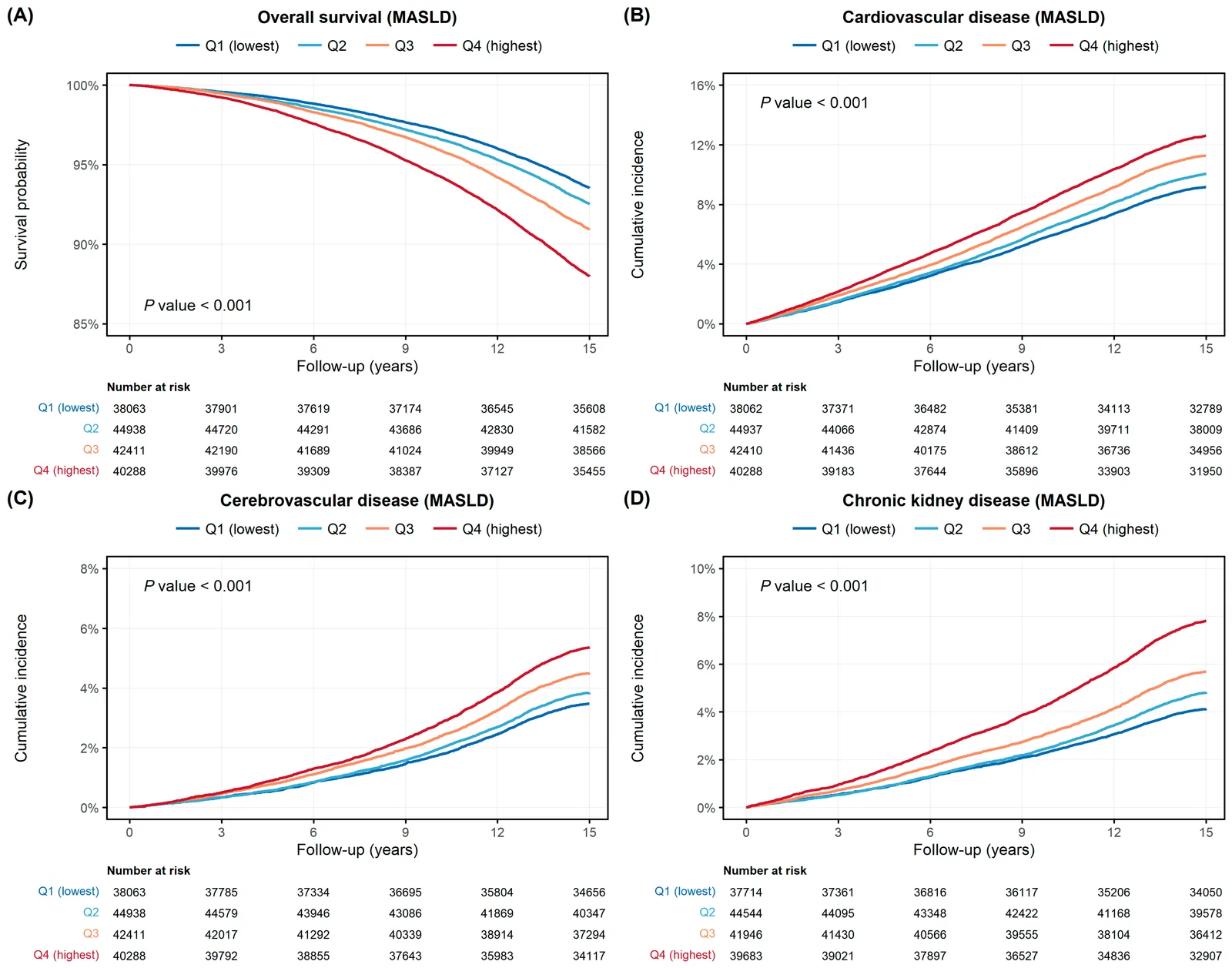
Event curves by RDW quartile in MASLD. Kaplan–Meier curve for overall survival (**A**) and cumulative incidence curves for cardiovascular disease (**B**), cerebrovascular disease (**C**), and chronic kidney disease (**D**) according to RDW quartile in the MASLD cohort. Number at risk tables are shown below each panel. RDW, red cell distribution width; MASLD, metabolic dysfunction-associated steatotic liver disease.

**Figure 4:**
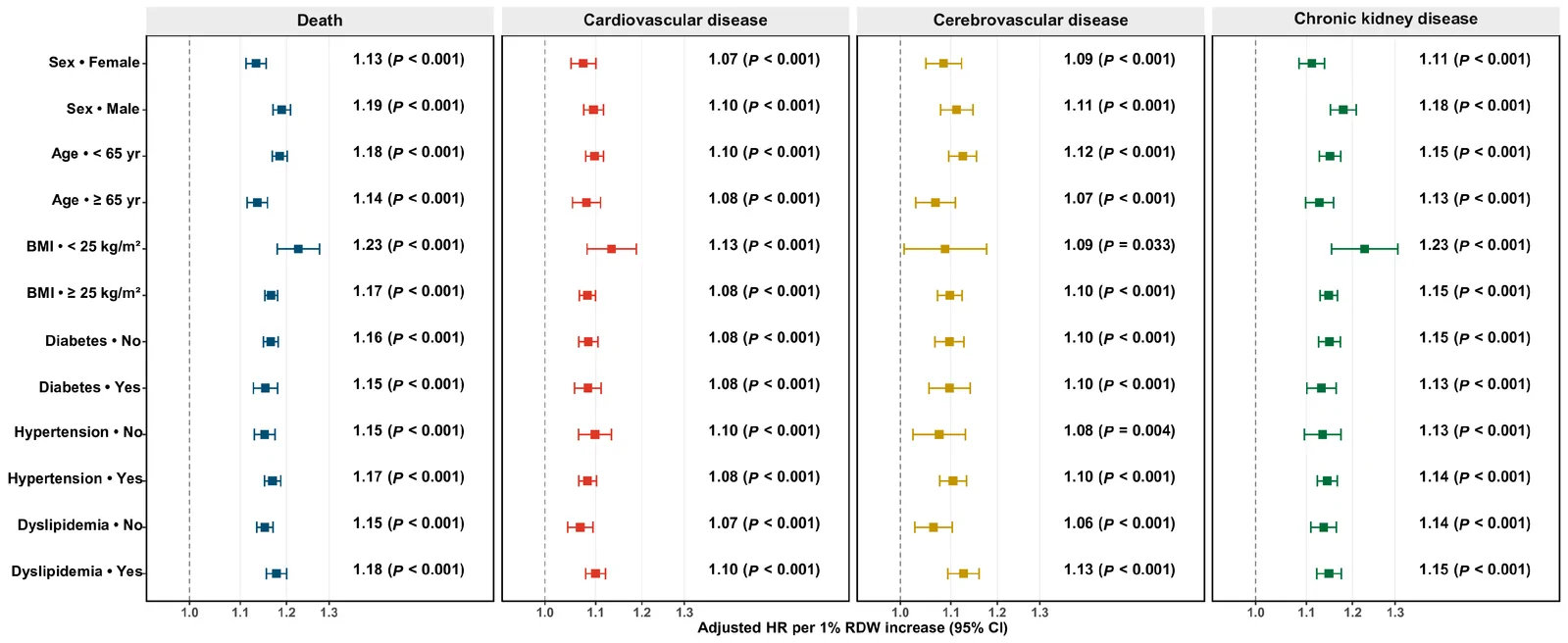
Subgroup analyses of RDW–outcome associations in MASLD. Forest plots of adjusted HRs per 1% increase in RDW across subgroups defined by sex, age, BMI, diabetes, hypertension, and dyslipidemia in the MASLD cohort. Dots indicate HRs and bars indicate 95% CIs. The vertical dashed line indicates an HR of 1.0 (null effect). RDW, red cell distribution width; MASLD, metabolic dysfunction-associated steatotic liver disease; BMI, body mass index; HR, hazard ratio; CI, confidence interval.

Kaplan–Meier and cumulative incidence curves in MASLD separated early and continued to diverge throughout follow-up. The Q4 curve showed the lowest overall survival and the highest cumulative incidence for cardiovascular disease, cerebrovascular disease, and chronic kidney disease; the log-rank *p* values were <0.001 for all four comparisons (Figure 3). The same visual gradient was observed in the total, No SLD, and MetALD cohorts (Supplementary Figures S1–S3). Predefined subgroup analyses in MASLD showed that the RDW association was present across sex, age, BMI, diabetes, hypertension, and dyslipidemia subgroups. For death, adjusted HRs per 1% RDW ranged from 1.13 in women to 1.23 in participants with BMI < 25 kg/m^2^. For cardiovascular disease, estimates ranged from 1.07 to 1.13; for cerebrovascular disease, from 1.06 to 1.13; and for chronic kidney disease, from 1.11 to 1.23 (Figure 4). Associations were directionally consistent across the total, No SLD, and MetALD cohorts, although the patterns were less pronounced outside the MASLD cohort (Supplementary Figures S4–S6).

### 3.4. Causal Interference Consistency

**Figure 5:**
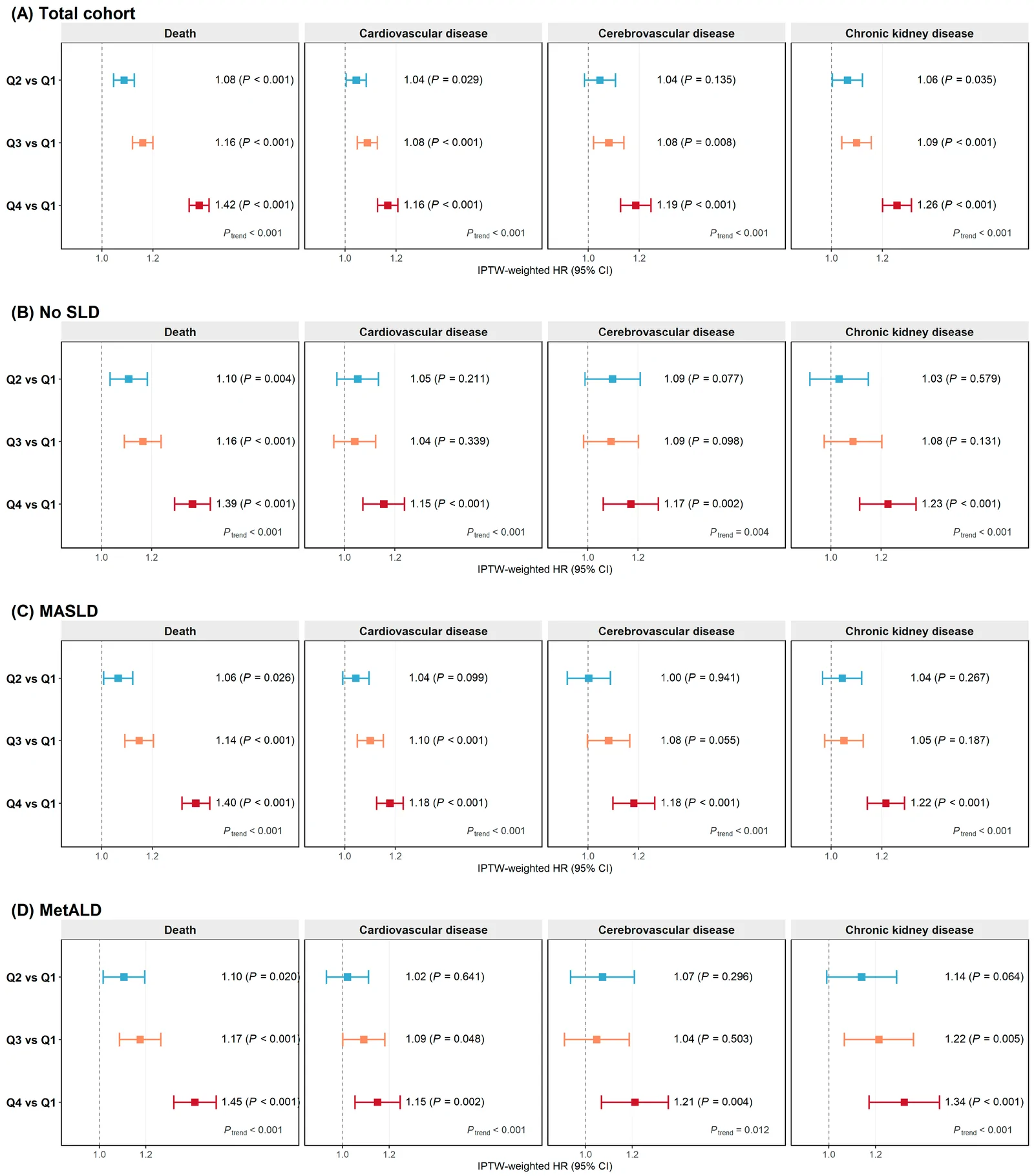
IPTW-weighted RDW quartile associations across the SLD spectrum. IPTW-weighted hazard ratios for RDW quartile contrasts in the total (**A**), No SLD (**B**), MASLD (**C**), and MetALD (**D**) cohorts. Q1 was used as the reference group. Dots indicate HRs and bars indicate 95% CIs. The vertical dashed line indicates an HR of 1.0 (null effect). IPTW, inverse probability of treatment weighting; RDW, red cell distribution width; SLD, steatotic liver disease; MASLD, metabolic dysfunction-associated steatotic liver disease; MetALD, MASLD with increased alcohol intake; HR, hazard ratio; CI, confidence interval.

A two-year landmark analysis excluding early events and early censoring showed maintained separation across RDW quartiles (Supplementary Figure S7). Competing risk analyses showed similar cumulative incidence patterns for non-mortality endpoints (Supplementary Figure S8). In IPTW analyses, covariate balance was achieved across all cohorts, with standardized mean differences < 0.10 after weighting (Supplementary Tables S7–S10 and Figure S9). Weighted Cox models showed persistent RDW quartile gradients. In the MASLD cohort, the IPTW-weighted HR for Q4 versus Q1 was 1.40 for death, 1.18 for cardiovascular disease, 1.18 for cerebrovascular disease, and 1.22 for chronic kidney disease (all *p*-trend < 0.001). Directionally consistent gradients were observed in the total, No SLD, and MetALD cohorts (Figure 5, Supplementary Table S11). IPTW-weighted event curves also showed maintained separation across RDW quartiles in all cohorts (Supplementary Figures S10–S13).

### 3.5. Mediation and Joint Marker Comparison

**Figure 6:**
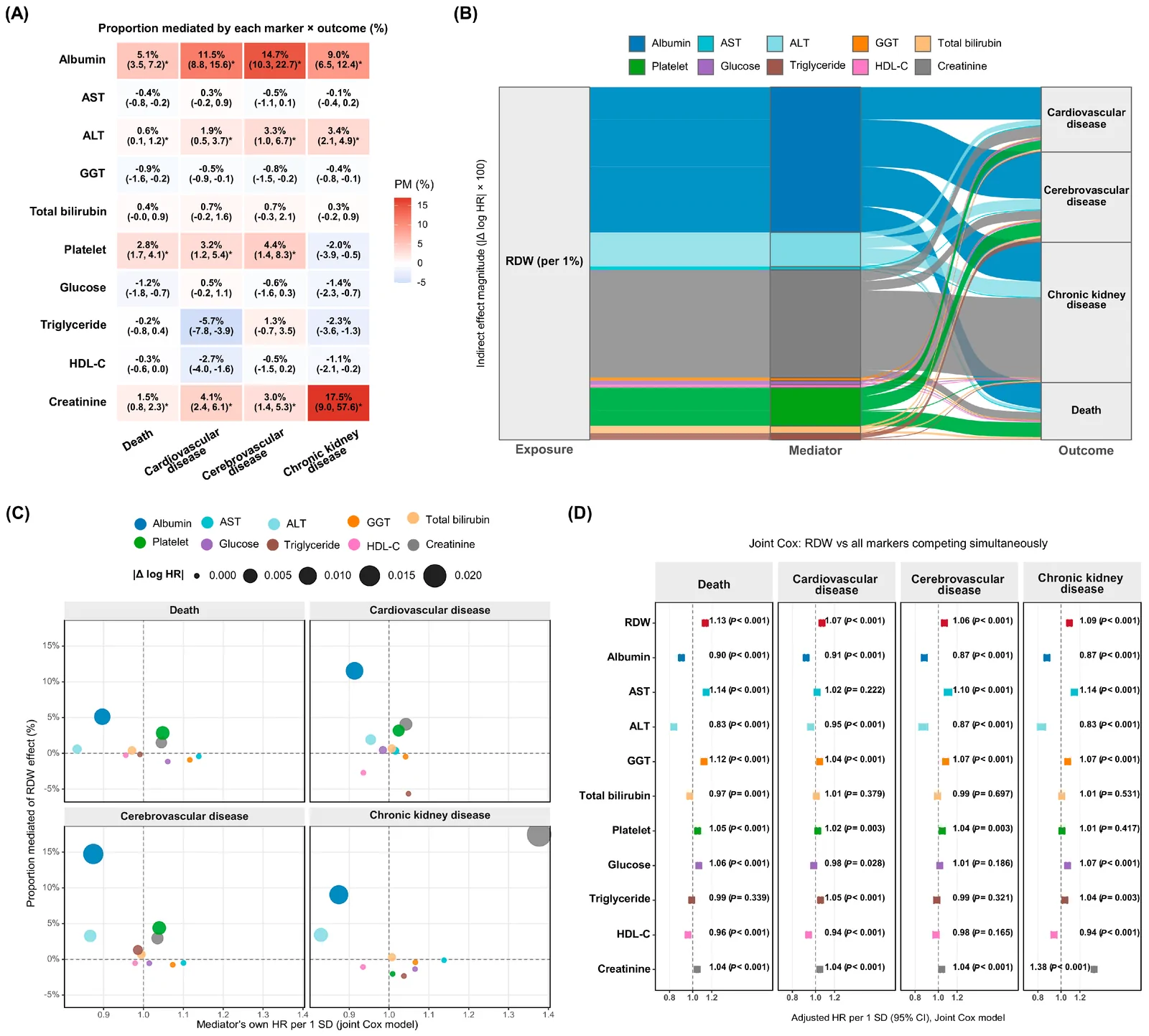
Mediation and joint marker comparison in MASLD. (**A**) Single-mediator proportions mediated for the RDW–outcome associations. (**B**) Sankey diagram showing indirect effects’ flow through the candidate biomarkers. (**C**) Bubble plot comparing mediator HRs and mediated proportions. (**D**) Joint Cox model including RDW and all candidate biomarkers simultaneously. Asterisks indicate statistical significance (*p* < 0.05). In panel (**C**), the vertical dashed line indicates an HR of 1.0. A positive mediated proportion indicates attenuation of the RDW–outcome association after accounting for the mediator, whereas a negative value indicates a suppressor pattern in which the direct association is strengthened. RDW, red cell distribution width; MASLD, metabolic dysfunction-associated steatotic liver disease; HR, hazard ratio; CI, confidence interval; AST, aspartate aminotransferase; ALT, alanine aminotransferase; GGT, gamma-glutamyl transferase; HDL-C, high-density lipoprotein cholesterol; SD, standard deviation.

Correlation analyses showed that RDW was associated with several candidate mediators, including albumin, liver enzymes, and creatinine, while these biomarkers also showed varying degrees of association with clinical outcomes (Supplementary Figure S14). Single-mediator analyses identified albumin as a consistent partial mediator across all four endpoints. The proportion mediated by albumin was 5.1% for death, 11.5% for cardiovascular disease, 14.7% for cerebrovascular disease, and 9.0% for chronic kidney disease. Creatinine mediated 17.5% of RDW’s association with chronic kidney disease but showed limited contributions to the other outcomes. AST, ALT, GGT, and total bilirubin showed minimal contributions when examined individually (Figure 6A–C and Supplementary Table S12).

Grouped mediation analyses showed that all 10 biomarkers jointly accounted for 11.6%, 14.4%, 28.5%, and 29.9% of the RDW association for death, cardiovascular disease, cerebrovascular disease, and chronic kidney disease, respectively. Among these, albumin showed consistent contributions across all four endpoints, whereas creatinine contributed primarily to chronic kidney disease. AST, ALT, GGT, and total bilirubin jointly accounted for 4.2%, 3.7%, 8.4%, and 6.5% of RDW’s association across the four outcomes (Supplementary Figure S15). Additional univariable analyses showed directionally consistent associations compared with multivariable models, although the effect sizes differed (Supplementary Figure S16). In joint Cox models including RDW and all 10 candidate mediators simultaneously, RDW remained independently associated with all outcomes, with HRs of 1.13 for death, 1.07 for cardiovascular disease, 1.06 for cerebrovascular disease, and 1.09 for chronic kidney disease (all *p* <0.001). Among the candidate mediators, albumin showed consistent inverse associations across all outcomes, while creatinine showed positive associations, particularly for chronic kidney disease. Other biomarkers showed smaller or inconsistent associations. (all *p* < 0.001; Figure 6D).

### 3.6. Noninvasive Tests for Fibrosis Score-Stratified Analyses

RDW quartiles were associated with a higher unadjusted proportion of participants in the high FIB-4 and high NFS categories (Supplementary Figure S17). In FIB-4-stratified Model 4 analyses, RDW remained associated with death across the low, indeterminate, and high FIB-4 categories (HRs 1.12, 1.15, and 1.20, respectively). Associations with non-mortality outcomes were observed in the low and indeterminate categories, whereas estimates in the high FIB-4 category remained directionally positive but were not statistically significant. NFS-stratified analyses showed the same pattern, with consistent death associations and positive non-mortality estimates across strata (Supplementary Figure S18, Tables S13 and S14).

## 4. Discussion

In this large cohort analysis of participants from the UK Biobank, including individuals with MASLD, baseline RDW was consistently associated with four clinically meaningful endpoints over a median follow-up of 13.6 years. Three observations merit emphasis. First, RDW showed a graded and independent association with all-cause mortality, cardiovascular disease, cerebrovascular disease, and chronic kidney disease after adjustment for demographic factors, metabolic comorbidities, and routine laboratory markers. The sequential adjustment models further illustrated the contribution of conventional risk factors to these associations. This sequential adjustment strategy was intended to assess whether the associations persisted after accounting for progressively broader sets of clinically relevant variables rather than to define a causal adjustment set. Effect estimates were attenuated after adjustment for age and sex and were further reduced after accounting for cardiometabolic factors, suggesting that part of the RDW-associated risk overlaps with demographic and metabolic risk profiles. Nevertheless, the associations persisted after additional adjustment for routine hepatic and renal laboratory parameters, supporting the significance of the observed associations to multivariable adjustment. In this context, “independent” refers to the persistence of the associations after adjustment for the covariates included in the multivariable models and does not imply biological or causal independence. Considering the relatively narrow distribution of RDW, the estimates per 1% absolute increase should be interpreted in the context of the observed RDW range, with the quartile analyses providing a complementary representation of the graded associations. Notably, the magnitude of the associations varied across outcomes, with stronger associations observed for all-cause mortality and chronic kidney disease than for cardiovascular and cerebrovascular outcomes. This distinction is significant, given the large sample size, in which even modest effect estimates may achieve statistical significance. Second, these associations were directionally consistent across the SLD spectrum and remained stable across multiple analytical approaches, including IPTW and landmark analyses. Third, only a modest proportion of the RDW signal was accounted for by conventional clinical biomarkers, even when considered jointly.

Prior studies have linked RDW to liver disease severity, but primarily in cross-sectional or disease-specific settings [17,18,23,24]. In contrast, the current analysis demonstrates that RDW identifies individuals at increased long-term risk for mortality, cardiovascular, cerebrovascular, and renal outcomes.

The biological basis of this signal is unlikely to be explained by hepatocellular injury alone. In the baseline analysis, higher RDW was not accompanied by higher aminotransferase or GGT levels, and grouped mediation analysis showed that AST, ALT, GGT, and total bilirubin accounted for only a small proportion of the association. Instead, RDW appears to reflect a broader physiological state. Mechanistically, RDW increases in the setting of impaired erythropoiesis, chronic inflammation, altered iron metabolism, and nutritional imbalance [11,25]. These processes overlap with MASLD’s pathophysiology but are not limited to liver-specific injury.

Albumin reflects not only hepatic synthetic function but also systemic inflammation and nutritional status, and its consistent association across outcomes suggests that RDW presents broader systemic factors beyond liver-specific processes [26,27]. In contrast, the role of creatinine is largely organ-specific and expected, reinforcing that RDW does not simply show renal dysfunction.

Notably, even when 10 routinely available biomarkers were considered jointly, a substantial proportion of the RDW association was not explained by these biomarkers. This indicates that RDW demonstrates aspects of systemic physiology that are not fully represented by conventional metabolic, hepatic, or renal measurements. Potential contributors include inflammatory cytokine activity, iron handling, oxidative stress, and muscle-related metabolic processes, all of which have been implicated in MASLD’s progression. Oxidative stress is regarded as one of the central mechanisms driving lipotoxicity, mitochondrial dysfunction, hepatocellular injury, and systemic metabolic dysregulation in MASLD [28]. Persistent oxidative stress can impair erythrocytes’ membrane integrity and shorten erythrocytes’ lifespan, and might therefore represent one of several biological mechanisms contributing to the observed association between higher RDW and adverse clinical outcomes [29,30]. However, oxidative stress was not directly measured in the present study, and the observed associations should not be interpreted as evidence that RDW specifically represents oxidative stress. These unmeasured domains would be particularly significant in MASLD, where multisystem dysregulation is a defining feature.

From a clinical perspective, these findings are clinically useful. RDW is universally available as part of the complete blood count, requires no additional testing, and incurs no incremental cost. In clinical settings where MASLD is first evaluated, risk stratification often depends on readily available laboratory data before advanced imaging or specialist referral. These findings support further investigation of RDW as a readily available marker associated with systemic risk in MASLD. However, the present analysis did not evaluate whether RDW improves discrimination, calibration, or clinical utility beyond established risk assessment tools, and dedicated prediction studies are required before its incremental value for risk stratification can be established. This is not to suggest that RDW should replace established noninvasive tests for fibrosis scores or cardiovascular risk models, but rather that it could provide additive information within routine clinical workflows. The strengths of the findings further support their interpretability. The associations were not materially altered after IPTW adjustment for measured confounding and persisted in the two-year landmark analysis excluding early events. This finding reduces the likelihood that the observed associations were driven solely by pre-existing or subclinical illness leading to both elevated baseline RDW and early clinical events, although reverse causation cannot be completely excluded. While residual confounding cannot be excluded in an observational study, the consistency of results across cohorts and analytical approaches supports the stability of the RDW signal without implying causality.

Several limitations should be acknowledged. First, as an observational study, causal inference cannot be established, and residual confounding remains possible despite extensive adjustment. In addition, RDW is influenced by hematological and nutritional factors, including anemia, altered erythropoiesis, iron or vitamin deficiencies, and other red-cell abnormalities that were not comprehensively accounted for in the present analysis. Therefore, residual confounding by these factors cannot be excluded, and RDW should be interpreted as a non-specific marker of systemic risk rather than a disease-specific biological marker. Second, UK Biobank participants are subject to healthy volunteer bias, which could affect generalizability. Third, MASLD was operationally defined using the FLI and cardiometabolic criteria rather than imaging or histology, introducing potential phenotype misclassification. In addition, because the FLI incorporates BMI, waist circumference, triglycerides, and GGT, some of which were also considered in subsequent analyses, structural overlap between the cohorts’ definition and analytical variables may have introduced additional dependencies and should be considered when interpreting the findings. Fourth, RDW was measured at a single time point, and longitudinal changes were not assessed. The mediation analyses were exploratory and associational, and causal interpretation would require strong assumptions regarding unmeasured confounding, including the absence of mediator–outcome confounding affected by the exposure. Accordingly, the mediated proportions should not be interpreted as evidence of causal pathways. Finally, direct biomarkers of oxidative stress were not available, precluding assessment of whether RDW specifically reflects oxidative stress in this population. RDW is also influenced by multiple inflammatory, hematologic, nutritional, and renal processes. Therefore, the biological mechanisms underlying the observed associations cannot be determined from the present study.

## 5. Conclusions

Higher RDW was consistently associated with adverse clinical outcomes in individuals with MASLD, and these associations persisted after adjustment for conventional liver and metabolic biomarkers. These findings suggest that RDW would represent systemic alterations not fully represented by routinely available clinical measures. Oxidative stress may represent one of several potential biological mechanisms underlying these associations, although this cannot be established from the present study. As a universally available and low-cost parameter, RDW might serve as a readily accessible marker associated with long-term systemic risk in individuals with MASLD. Further studies are required to determine its incremental predictive value and clinical utility for risk stratification.

## Figures and Tables

**Table 1:** Baseline characteristics of the MASLD cohort by RDW quartile.

Characteristic	Overall N = 165,700 ^1^	Q1 N = 38,063 ^1^	Q2 N = 44,938 ^1^	Q3 N = 42,411 ^1^	Q4 N = 40,288 ^1^	*p* Value ^2^
Age, years	56.2 ± 8.0	54.9 ± 8.1	55.9 ± 8.0	56.8 ± 7.9	57.1 ± 7.9	<0.001
Male	96,166 (58.0%)	22,771 (59.8%)	27,574 (61.4%)	25,240 (59.5%)	20,581 (51.1%)	<0.001
BMI (kg/m^2^)	29.6 ± 4.3	29.0 ± 3.8	29.3 ± 4.0	29.6 ± 4.3	30.5 ± 5.0	<0.001
Waist circumference (cm)	97.0 ± 10.6	95.6 ± 9.7	96.5 ± 10.2	97.3 ± 10.5	98.3 ± 11.5	<0.001
Smoking status						<0.001
Never	93,156 (56.2%)	21,868 (57.5%)	25,434 (56.6%)	23,713 (55.9%)	22,141 (55.0%)	
Previous	56,208 (33.9%)	13,263 (34.8%)	15,453 (34.4%)	14,323 (33.8%)	13,169 (32.7%)	
Current	16,336 (9.9%)	2932 (7.7%)	4051 (9.0%)	4375 (10.3%)	4978 (12.4%)	
Diabetes	21,736 (13.1%)	4331 (11.4%)	5444 (12.1%)	5380 (12.7%)	6581 (16.3%)	<0.001
Hypertension	78,356 (47.3%)	16,794 (44.1%)	20,504 (45.6%)	20,239 (47.7%)	20,819 (51.7%)	<0.001
Dyslipidemia	52,328 (31.6%)	11,513 (30.2%)	13,941 (31.0%)	13,563 (32.0%)	13,311 (33.0%)	<0.001
Glucose (mg/dL)	93.3 ± 23.9	93.9 ± 26.3	93.2 ± 23.5	92.7 ± 21.7	93.7 ± 24.0	<0.001
Triglyceride (mg/dL)	186.1 ± 96.1	196.0 ± 101.8	189.8 ± 97.6	184.3 ± 93.8	174.3 ± 89.7	<0.001
HDL-C (mg/dL)	50.6 ± 11.9	49.8 ± 11.4	50.2 ± 11.6	50.8 ± 11.9	51.5 ± 12.5	<0.001
Creatinine (mg/dL)	0.8 ± 0.2	0.8 ± 0.2	0.8 ± 0.2	0.9 ± 0.2	0.8 ± 0.3	<0.001
AST (U/L)	27.0 ± 10.3	27.3 ± 9.6	27.1 ± 9.6	26.8 ± 9.7	26.6 ± 12.0	<0.001
ALT (U/L)	26.7 ± 15.0	28.0 ± 15.3	27.4 ± 15.1	26.5 ± 14.9	25.1 ± 14.6	<0.001
GGT (U/L)	42.2 ± 39.7	43.4 ± 38.4	42.3 ± 38.2	41.7 ± 39.1	41.3 ± 42.9	<0.001
Albumin, (g/dL)	4.5 ± 0.3	4.6 ± 0.3	4.5 ± 0.3	4.5 ± 0.3	4.5 ± 0.3	<0.001
Platelet (10^9^/L)	254.4 ± 60.0	250.3 ± 55.6	251.4 ± 56.2	252.3 ± 57.5	263.7 ± 68.9	<0.001
Total bilirubin (mg/dL)	0.5 ± 0.3	0.6 ± 0.3	0.5 ± 0.3	0.5 ± 0.2	0.5 ± 0.2	<0.001
RDW (%)	13.5 ± 0.9	12.6 ± 0.3	13.1 ± 0.1	13.6 ± 0.1	14.6 ± 1.1	<0.001

^1^ Mean ± SD; n (%). ^2^ One-way analysis of the means (not assuming equal variances); Pearson’s chi-squared test. Values are the mean ± SD for continuous variables and n (%) for categorical variables. The *p* values are from one-way ANOVA for continuous variables and chi-squared tests for categorical variables across RDW quartiles. RDW quartiles were defined in the analytic cohort. RDW, red cell distribution width; BMI, body mass index; AST, aspartate aminotransferase; ALT, alanine aminotransferase; GGT, gamma-glutamyl transferase; HDL-C, high-density lipoprotein cholesterol.

**Table 2:** Cox proportional hazards models for RDW quartiles and clinical outcomes in the MASLD cohort.

Outcome	Form	Model 1	Model 2	Model 3	Model 4
Death	Q1 (ref)	1.00 (ref)	1.00 (ref)	1.00 (ref)	1.00 (ref)
	Q2 vs. Q1	1.19 (1.13–1.25) *p* < 0.001	1.19 (1.13–1.25) *p* < 0.001	1.08 (1.03–1.13) *p* = 0.002	1.06 (1.01–1.11) *p* = 0.025
	Q3 vs. Q1	1.44 (1.37–1.51) *p* < 0.001	1.44 (1.37–1.51) *p* < 0.001	1.22 (1.16–1.28) *p* < 0.001	1.16 (1.10–1.22) *p* < 0.001
	Q4 vs. Q1	1.93 (1.84–2.02) *p* < 0.001	1.93 (1.84–2.02) *p* < 0.001	1.62 (1.54–1.69) *p* < 0.001	1.45 (1.38–1.52) *p* < 0.001
	*p*-trend	*p* < 0.001	*p* < 0.001	*p* < 0.001	*p* < 0.001
Cardiovascular disease	Q1 (ref)	1.00 (ref)	1.00 (ref)	1.00 (ref)	1.00 (ref)
	Q2 vs. Q1	1.11 (1.06–1.16) *p* < 0.001	1.11 (1.06–1.16) *p* < 0.001	1.04 (0.99–1.08) *p* = 0.125	1.03 (0.99–1.08) *p* = 0.172
	Q3 vs. Q1	1.25 (1.19–1.30) *p* < 0.001	1.25 (1.19–1.30) *p* < 0.001	1.12 (1.07–1.17) *p* < 0.001	1.11 (1.06–1.16) *p* < 0.001
	Q4 vs. Q1	1.41 (1.35–1.48) *p* < 0.001	1.41 (1.35–1.48) *p* < 0.001	1.29 (1.24–1.35) *p* < 0.001	1.21 (1.16–1.26) *p* < 0.001
	*p*-trend	*p* < 0.001	*p* < 0.001	*p* < 0.001	*p* < 0.001
Cerebrovascular disease	Q1 (ref)	1.00 (ref)	1.00 (ref)	1.00 (ref)	1.00 (ref)
	Q2 vs. Q1	1.11 (1.03–1.19) *p* = 0.005	1.11 (1.03–1.19) *p* = 0.005	1.01 (0.94–1.09) *p* = 0.736	1.01 (0.94–1.08) *p* = 0.836
	Q3 vs. Q1	1.30 (1.21–1.40) *p* < 0.001	1.30 (1.21–1.40) *p* < 0.001	1.11 (1.03–1.19) *p* = 0.005	1.09 (1.02–1.17) *p* = 0.014
	Q4 vs. Q1	1.57 (1.47–1.68) *p* < 0.001	1.57 (1.47–1.68) *p* < 0.001	1.32 (1.23–1.41) *p* < 0.001	1.23 (1.15–1.32) *p* < 0.001
	*p*-trend	*p* < 0.001	*p* < 0.001	*p* < 0.001	*p* < 0.001
Chronic kidney disease	Q1 (ref)	1.00 (ref)	1.00 (ref)	1.00 (ref)	1.00 (ref)
	Q2 vs. Q1	1.17 (1.10–1.25) *p* < 0.001	1.17 (1.10–1.25) *p* < 0.001	1.07 (1.00–1.14) *p* = 0.039	1.06 (0.99–1.13) *p* = 0.095
	Q3 vs. Q1	1.40 (1.31–1.49) *p* < 0.001	1.40 (1.31–1.49) *p* < 0.001	1.18 (1.11–1.26) *p* < 0.001	1.15 (1.08–1.23) *p* < 0.001
	Q4 vs. Q1	1.94 (1.82–2.06) *p* < 0.001	1.94 (1.82–2.06) *p* < 0.001	1.59 (1.50–1.69) *p* < 0.001	1.42 (1.34–1.52) *p* < 0.001
	*p*-trend	*p* < 0.001	*p* < 0.001	*p* < 0.001	*p* < 0.001

Hazard ratios (HR) with 95% confidence intervals (CI) from Cox proportional hazards models comparing RDW quartiles (Q1 = reference). Model 1: crude. Model 2: adjusted for age and sex. Model 3: Model 2 + BMI, diabetes, hypertension, dyslipidemia, and smoking status. Model 4: Model 3 + AST, ALT, GGT, platelet count, albumin, and creatinine. Previous events at baseline were excluded from each outcome analysis. The *p*-trend was tested by modelling RDW quartile as an ordinal variable. RDW, red cell distribution width; AST, aspartate aminotransferase; ALT, alanine aminotransferase; GGT, gamma-glutamyl transferase.

## Data Availability

The data used in this study are available from the UK Biobank (Application No. 117214). Restrictions apply to the availability of these data, which were used under license for the current study and are therefore not publicly available. Researchers may apply for access to the UK Biobank resource at https://www.ukbiobank.ac.uk/ (accessed on 1 June 2026).

## Supplementary Materials

The following supporting information can be downloaded at https://www.mdpi.com/article/10.3390/antiox15091065/s1. Supplementary Figure S1. Event curves by RDW quartile in the Total cohort. Supplementary Figure S2. Event curves by RDW quartile in the No SLD cohort. Supplementary Figure S3. Event curves by RDW quartile in the MetALD cohort. Supplementary Figure S4. Subgroup analyses of RDW-outcome associations in the Total cohort. Supplementary Figure S5. Subgroup analyses of RDW-outcome associations in the No SLD cohort. Supplementary Figure S6. Subgroup analyses of RDW-outcome associations in the MetALD cohort. Supplementary Figure S7. Two-year landmark analysis by RDW quartile. Supplementary Figure S8. Competing-risk cumulative-incidence analyses. Supplementary Figure S9. Covariate balance after IPTW. Supplementary Figure S10. IPTW-weighted event curves in the Total cohort. Supplementary Figure S11. IPTW-weighted event curves in the No SLD cohort. Supplementary Figure S12. IPTW-weighted event curves in the MASLD cohort. Supplementary Figure S13. IPTW-weighted event curves in the MetALD cohort. Supplementary Figure S14. Correlation structure among RDW, candidate biomarkers, and outcomes. Supplementary Figure S15. Grouped mediation analysis in MASLD. Supplementary Figure S16. Single-marker Cox analyses in MASLD. Supplementary Figure S17. Distribution of fibrosis-score categories by RDW quartile. Supplementary Figure S18. RDW-outcome associations stratified by fibrosis-score category. Supplementary Table S1. Baseline characteristics of the total cohort by RDW quartile. Supplementary Table S2. Baseline characteristics of the no SLD cohort by RDW quartile. Supplementary Table S3. Baseline characteristics of the MetALD cohort by RDW quartile. Supplementary Table S4. Cox proportional hazards models for RDW quartiles and clinical outcomes in the total cohort. Supplementary Table S5. Cox proportional hazards models for RDW quartiles and clinical outcomes in the no SLD cohort. Supplementary Table S6. Cox proportional hazards models for RDW quartiles and clinical outcomes in the MetALD cohort. Supplementary Table S7. Baseline characteristics of the total cohort by RDW quartile after IPTW weighting. Supplementary Table S8. Baseline characteristics of the no SLD cohort by RDW quartile after IPTW weighting. Supplementary Table S9. Baseline characteristics of the MASLD cohort by RDW quartile after IPTW weighting. Supplementary Table S10. Baseline characteristics of the MetALD cohort by RDW quartile after IPTW weighting. Supplementary Table S11. IPTW-weighted Cox regression of RDW quartile on clinical outcomes. Supplementary Table S12. Mediation and joint Cox marker comparison in the MASLD cohort. Supplementary Table S13. Cox regression of RDW per 1% across clinical outcomes, stratified by FIB-4 category in the MASLD cohort. Supplementary Table S14. Cox regression of RDW per 1% across clinical outcomes, stratified by NFS category in the MASLD cohort.

## Abbreviations

The following abbreviations are used in this manuscript.

ALT	Alanine aminotransferase
AST	Aspartate aminotransferase
BMI	Body mass index
CI	Confidence interval
FIB-4	Fibrosis-4 index
FLI	Fatty liver index
GGT	Gamma-glutamyl transferase
HDL	High-density lipoprotein
HR	Hazard ratio
ICD-10	International Classification of Diseases, 10th Revision
IPTW	Inverse probability of treatment weighting
MASLD	Metabolic dysfunction-associated steatotic liver disease
MetALD	Metabolic dysfunction-associated steatotic liver disease with increased alcohol intake
NAFLD	Non-alcoholic fatty liver disease
NFS	NAFLD fibrosis score
RDW	Red cell distribution width
SLD	Steatotic liver disease
