# Supplementary material for: Red Cell Distribution Width as an Independent Prognostic Biomarker in MASLD
Source: Antioxidants (Basel). 2026 Aug 25;15(9):1065. doi: 10.3390/antiox15091065 (PMC13603125; doi:10.3390/antiox15091065)
Supplement: Supplementary file 1 [file antioxidants-15-01065-s001.zip › antioxidants-4481688-supplementary.pdf]

Supplementary Information

Red cell distribution width as an independent prognostic biomarker in MASLD

Tom Ryu[1], Jaejun Lee[2], Ji Won Han[2], Hyun Yang[2] and Keungmo Yang[2]

[1]Department of Internal Medicine, Institute for Digestive Research, Digestive Disease Center, Soonchunhyang University College of Medicine, Seoul 04401, Republic of Korea
[2]Department of Internal Medicine, Division of Gastroenterology and Hepatology, College of Medicine, The Catholic University of Korea, Seoul 06591, Republic of Korea

ADDRESS CORRESPONDENCE AND REPRINT REQUESTS TO:
Keungmo Yang, M.D., Ph.D., Department of Internal Medicine, Division of Gastroenterology and Hepatology, College of Medicine, The Catholic University of Korea, Seoul 06591, Republic of Korea. Tel:+82-2-2258-2073 FAX:+82-2-3481-4025 E-mail: yang27jin@catholic.ac.kr

Supplementary Figures

- **Supplementary Fig. 1.** Event curves by RDW quartile in the Total cohort.
- **Supplementary Fig. 2.** Event curves by RDW quartile in the No SLD cohort.
- **Supplementary Fig. 3.** Event curves by RDW quartile in the MetALD cohort.
- **Supplementary Fig. 4.** Subgroup analyses of RDW-outcome associations in the Total cohort.
- **Supplementary Fig. 5.** Subgroup analyses of RDW-outcome associations in the No SLD cohort.
- **Supplementary Fig. 6.** Subgroup analyses of RDW-outcome associations in the MetALD cohort
- **Supplementary Fig. 7.** Two-year landmark analysis by RDW quartile.
- **Supplementary Fig. 8.** Competing-risk cumulative-incidence analyses.
- **Supplementary Fig. 9.** Covariate balance after IPTW.
- **Supplementary Fig. 10.** IPTW-weighted event curves in the Total cohort,
- **Supplementary Fig. 11.** IPTW-weighted event curves in the No SLD cohort.
- **Supplementary Fig. 12.** IPTW-weighted event curves in the MASLD cohort.
- **Supplementary Fig. 13.** IPTW-weighted event curves in the MetALD cohort.
- **Supplementary Fig. 14.** Correlation structure among RDW, candidate biomarkers, and outcomes.
- **Supplementary Fig. 15.** Grouped mediation analysis in MASLD.
- **Supplementary Fig. 16.** Single-marker Cox analyses in MASLD.
- **Supplementary Fig. 17.** Distribution of fibrosis-score categories by RDW quartile.
- **Supplementary Fig. 18.** RDW-outcome associations stratified by fibrosis-score category.

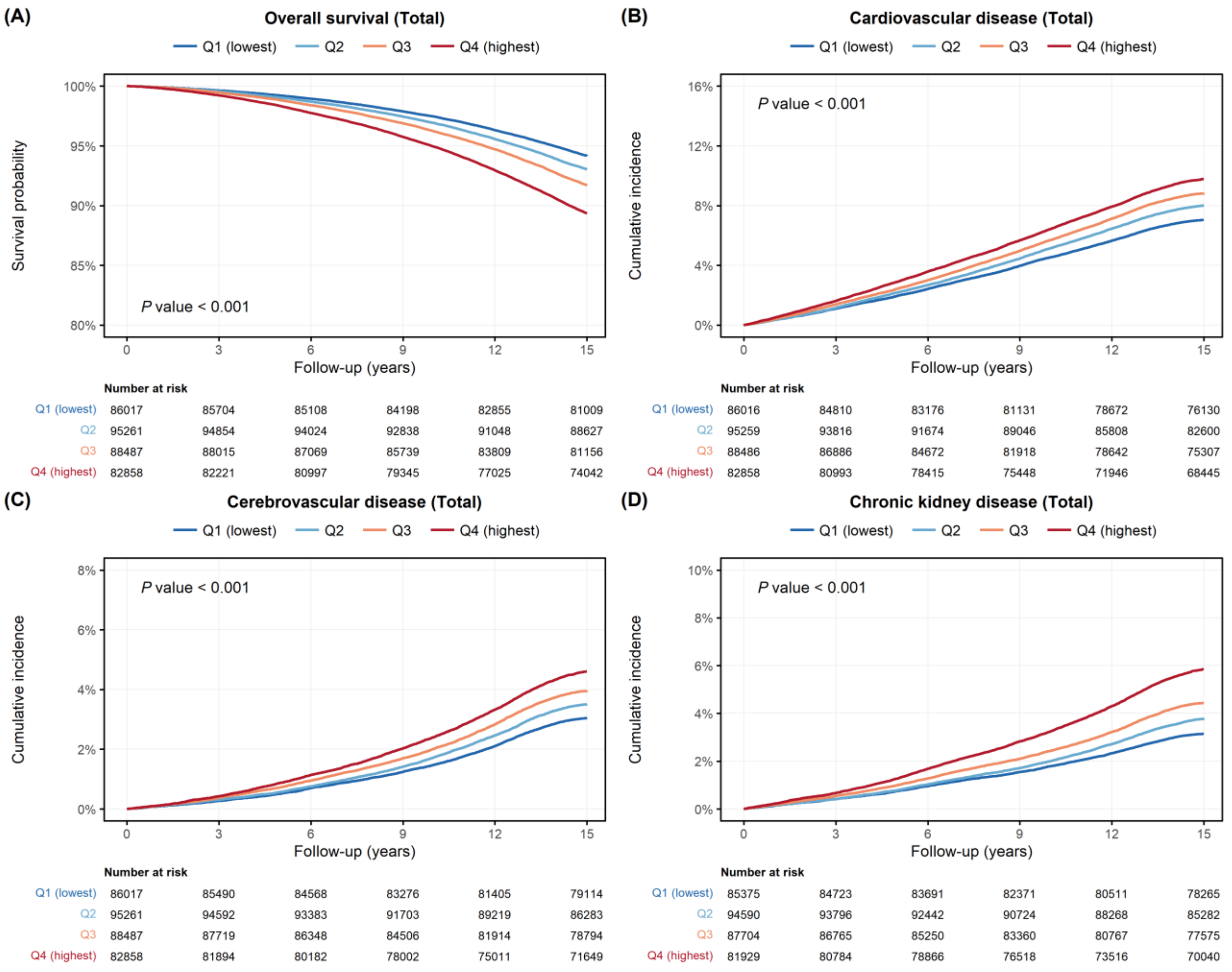

Supplementary Fig. 1. Event curves by RDW quartile in the Total cohort. Kaplan-Meier curve for overall survival (A) and cumulative-incidence curves for cardiovascular disease (B), cerebrovascular disease (C), and chronic kidney disease (D) according to RDW quartile in the Total cohort. RDW, red cell distribution width.

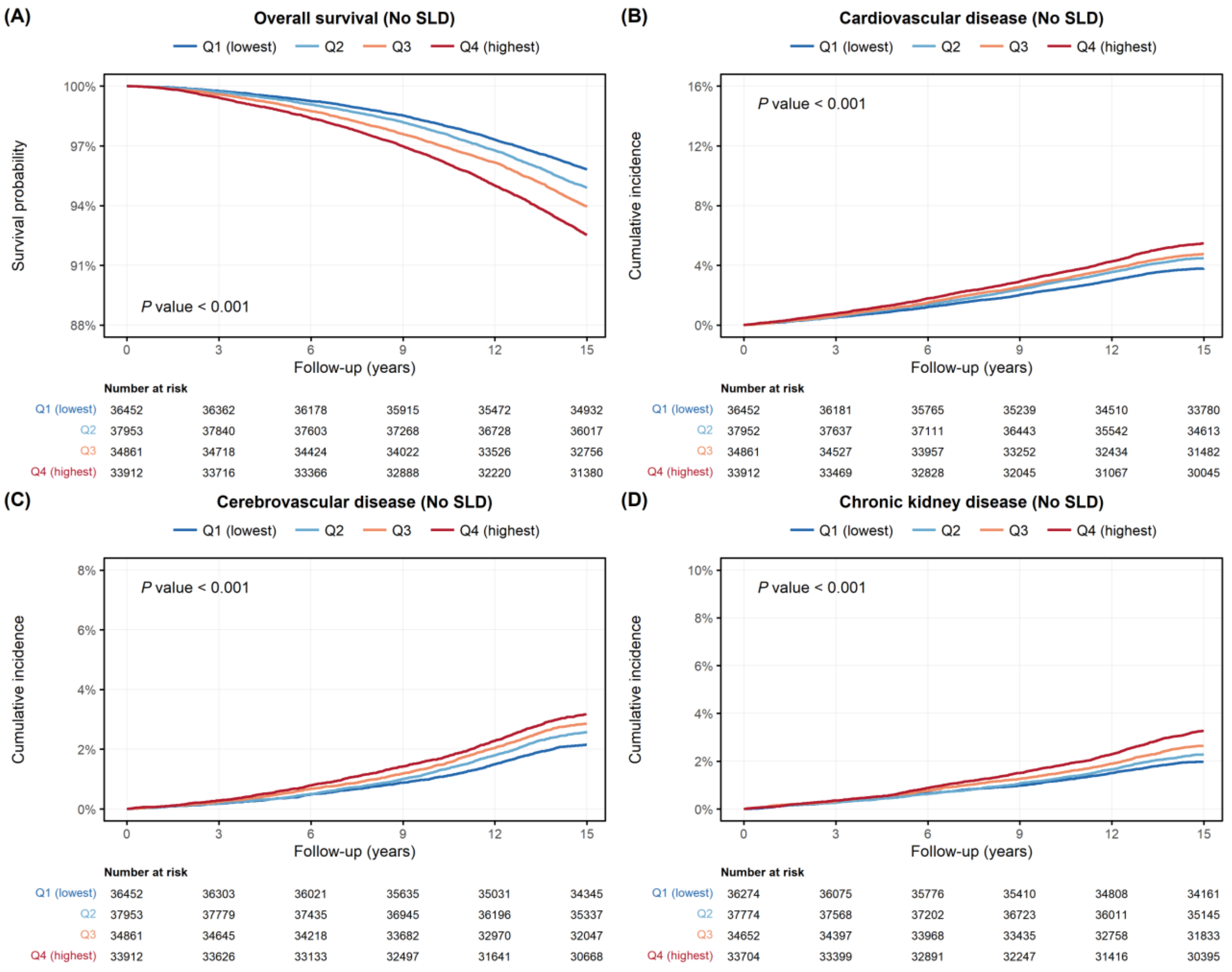

Supplementary Fig. 2. Event curves by RDW quartile in the No SLD cohort. Kaplan-Meier curve for overall survival (A) and cumulative-incidence curves for cardiovascular disease (B), cerebrovascular disease (C), and chronic kidney disease (D) according to RDW quartile in participants without steatotic liver disease. RDW, red cell distribution width; SLD, steatotic liver disease.

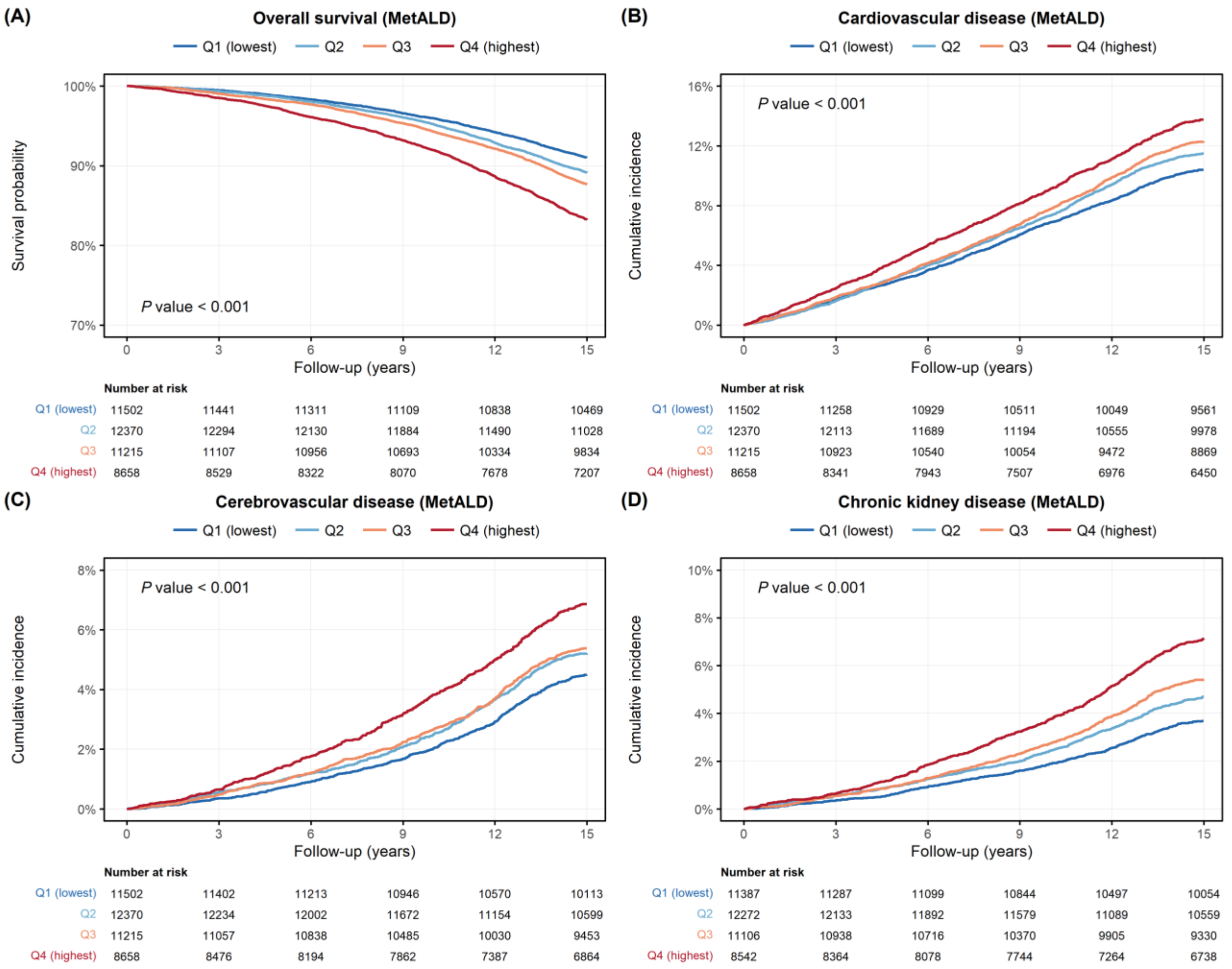

Supplementary Fig. 3. Event curves by RDW quartile in the MetALD cohort. Kaplan-Meier curve for overall survival and cumulative-incidence curves for cardiovascular disease, cerebrovascular disease, and chronic kidney disease according to RDW quartile in the MetALD cohort. RDW, red cell distribution width; MetALD, MASLD with increased alcohol intake.

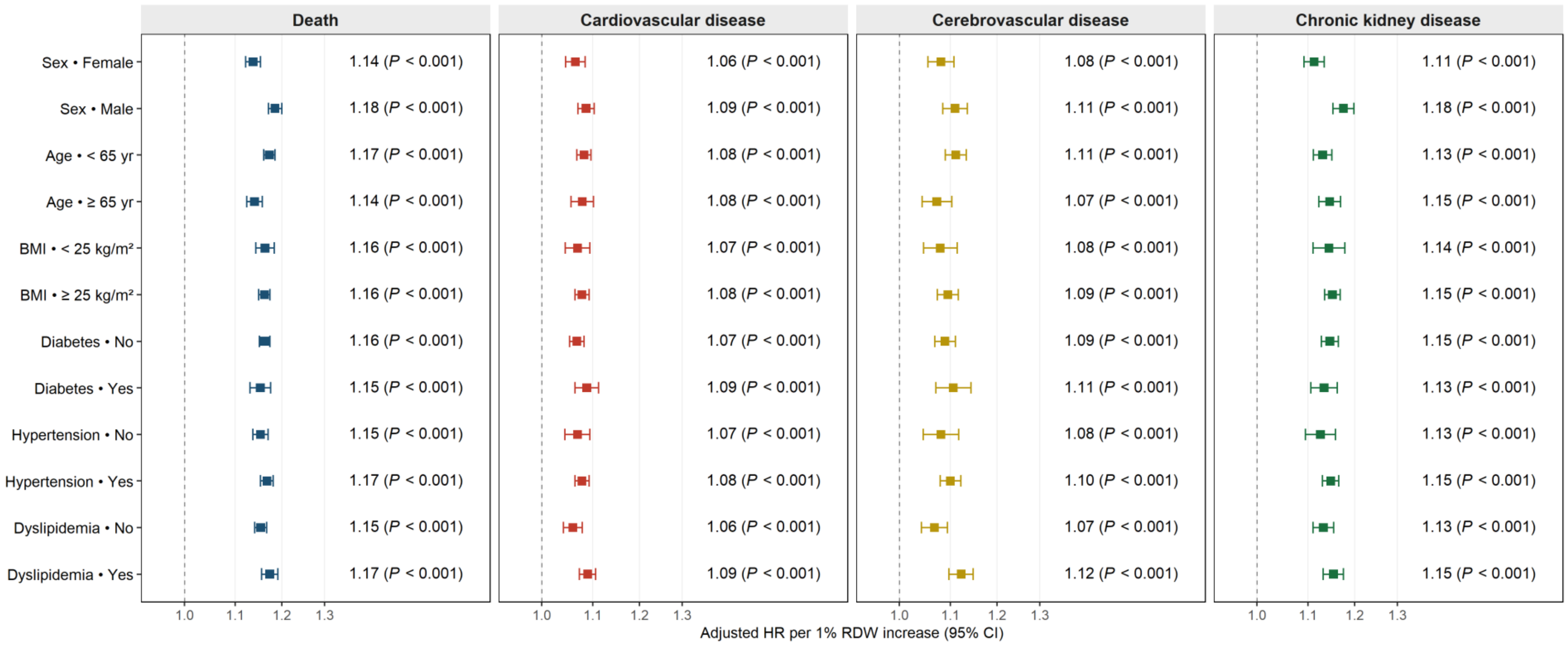

Supplementary Fig. 4. Subgroup analyses of RDW-outcome associations in the Total cohort. Subgroup-specific adjusted HRs per 1% increase in RDW according to sex, age, BMI, diabetes, hypertension, and dyslipidemia in the Total cohort. RDW, red cell distribution width; BMI, body mass index; HR, hazard ratio; CI, confidence interval.

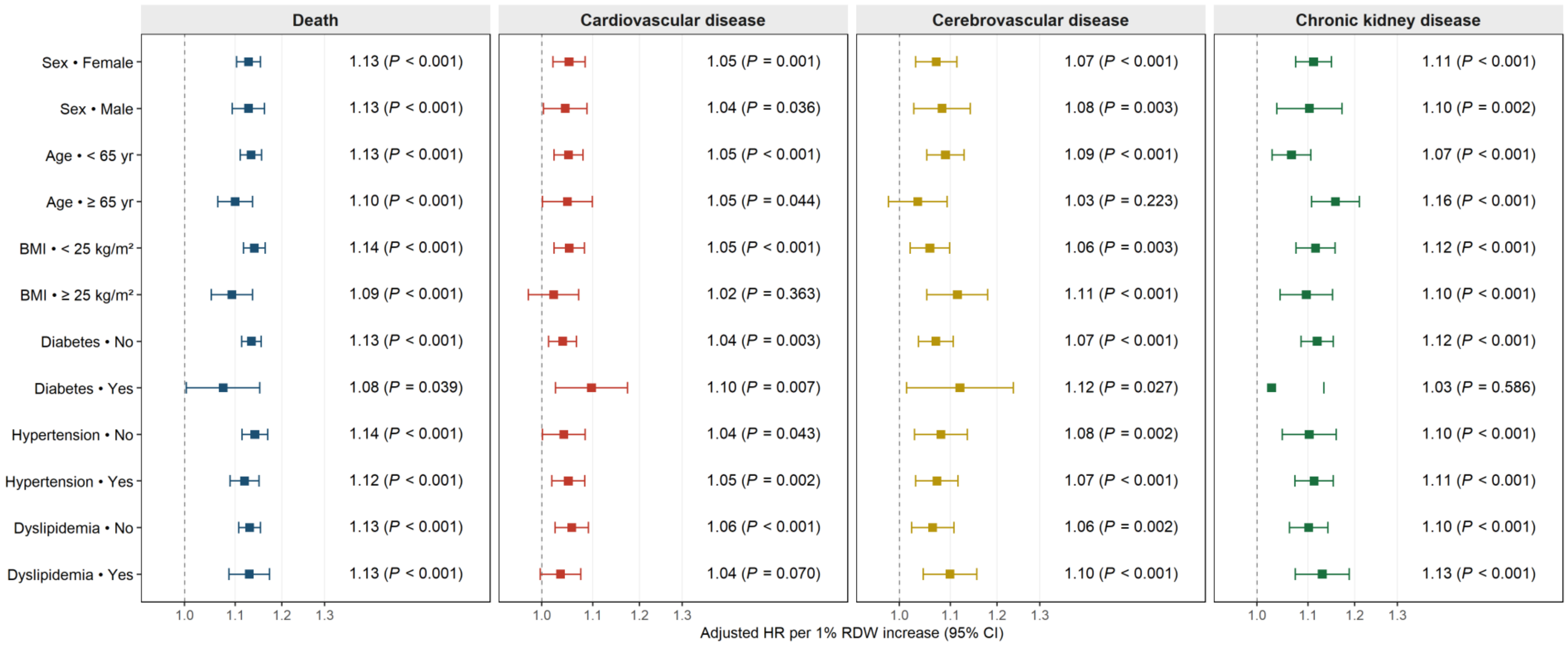

Supplementary Fig. 5. Subgroup analyses of RDW-outcome associations in the No SLD cohort. Subgroup-specific adjusted HRs per 1% increase in RDW according to sex, age, BMI, diabetes, hypertension, and dyslipidemia in the No SLD cohort. RDW, red cell distribution width; SLD, steatotic liver disease; BMI, body mass index; HR, hazard ratio; CI, confidence interval.

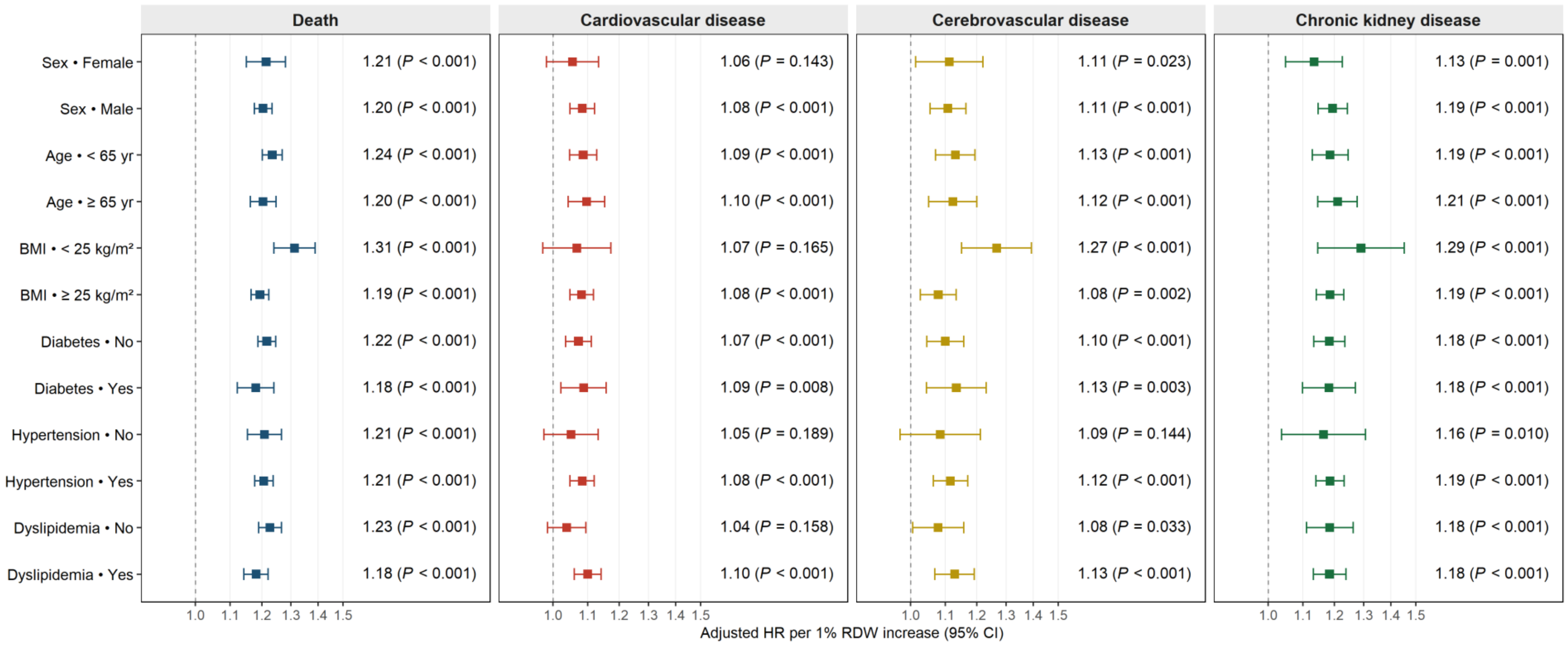

Supplementary Fig. 6. Subgroup analyses of RDW-outcome associations in the MetALD cohort. Subgroup-specific adjusted HRs per 1% increase in RDW according to sex, age, BMI, diabetes, hypertension, and dyslipidemia in the MetALD cohort. RDW, red cell distribution width; MetALD, MASLD with increased alcohol intake; BMI, body mass index; HR, hazard ratio; CI, confidence interval.

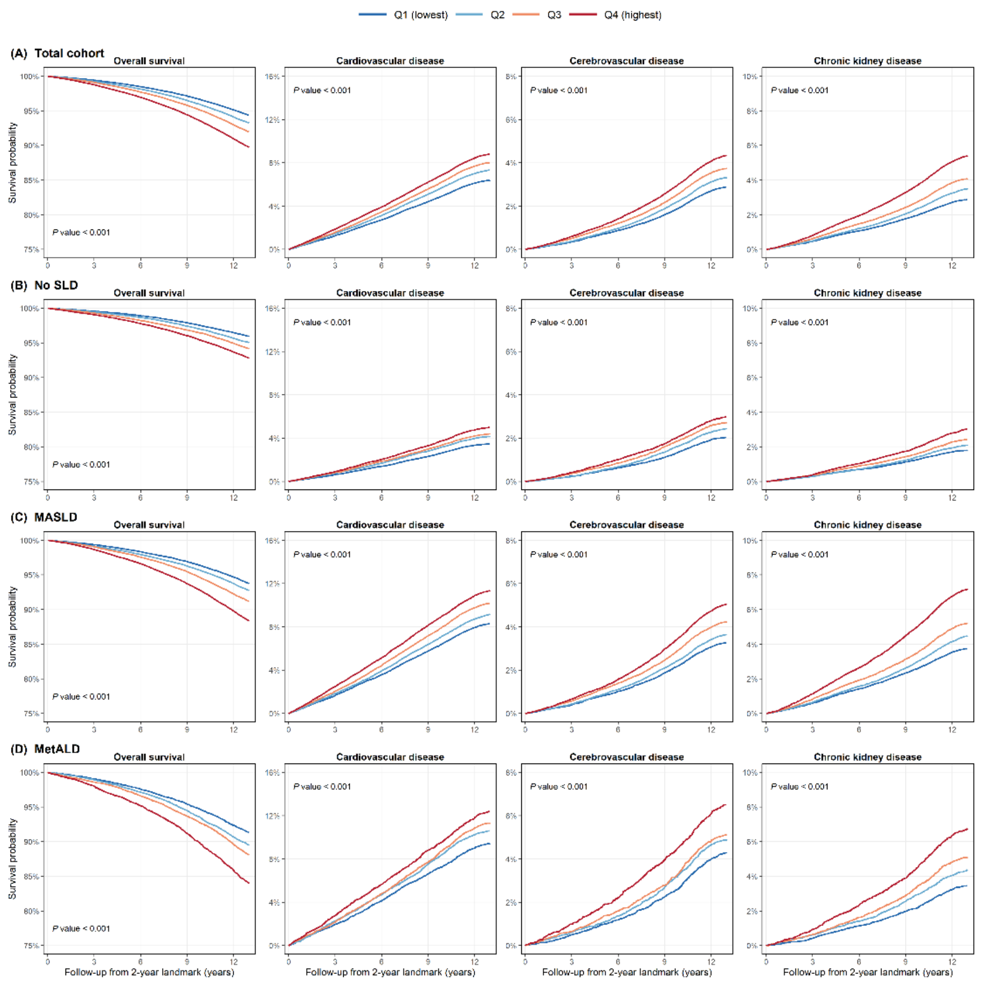

Supplementary Fig. 7. Two-year landmark analysis by RDW quartile. Kaplan-Meier and cumulative-incidence curves after excluding participants with events or censoring within the first two years of follow-up. Curves are shown across the Total, No SLD, MASLD, and MetALD cohorts. RDW, red cell distribution width; SLD, steatotic liver disease; MASLD, metabolic dysfunction-associated steatotic liver disease; MetALD, MASLD with increased alcohol intake.

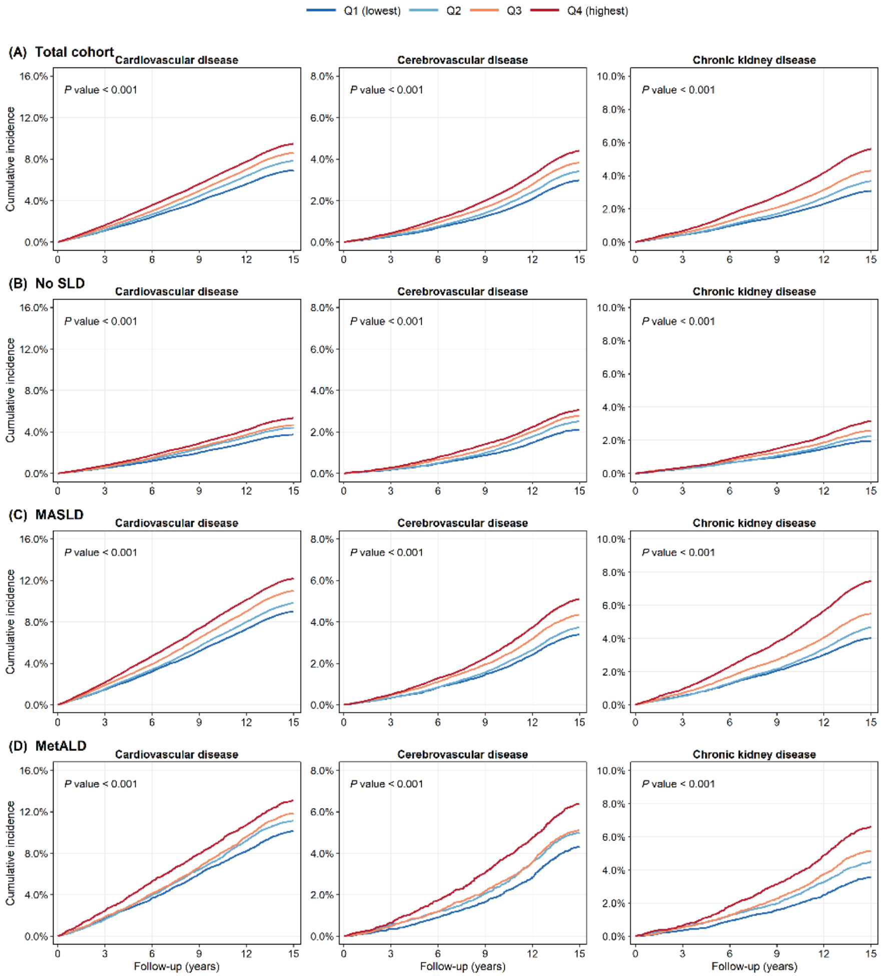

Supplementary Fig. 8. Competing-risk cumulative-incidence analyses. Cumulative-incidence curves for cardiovascular disease, cerebrovascular disease, and chronic kidney disease by RDW quartile, treating all-cause death as a competing event. Curves are shown across the Total (A), No SLD (B), MASLD (C), and MetALD (D) cohorts. RDW, red cell distribution width; SLD, steatotic liver disease; MASLD, metabolic dysfunction-associated steatotic liver disease; MetALD, MASLD with increased alcohol intake.

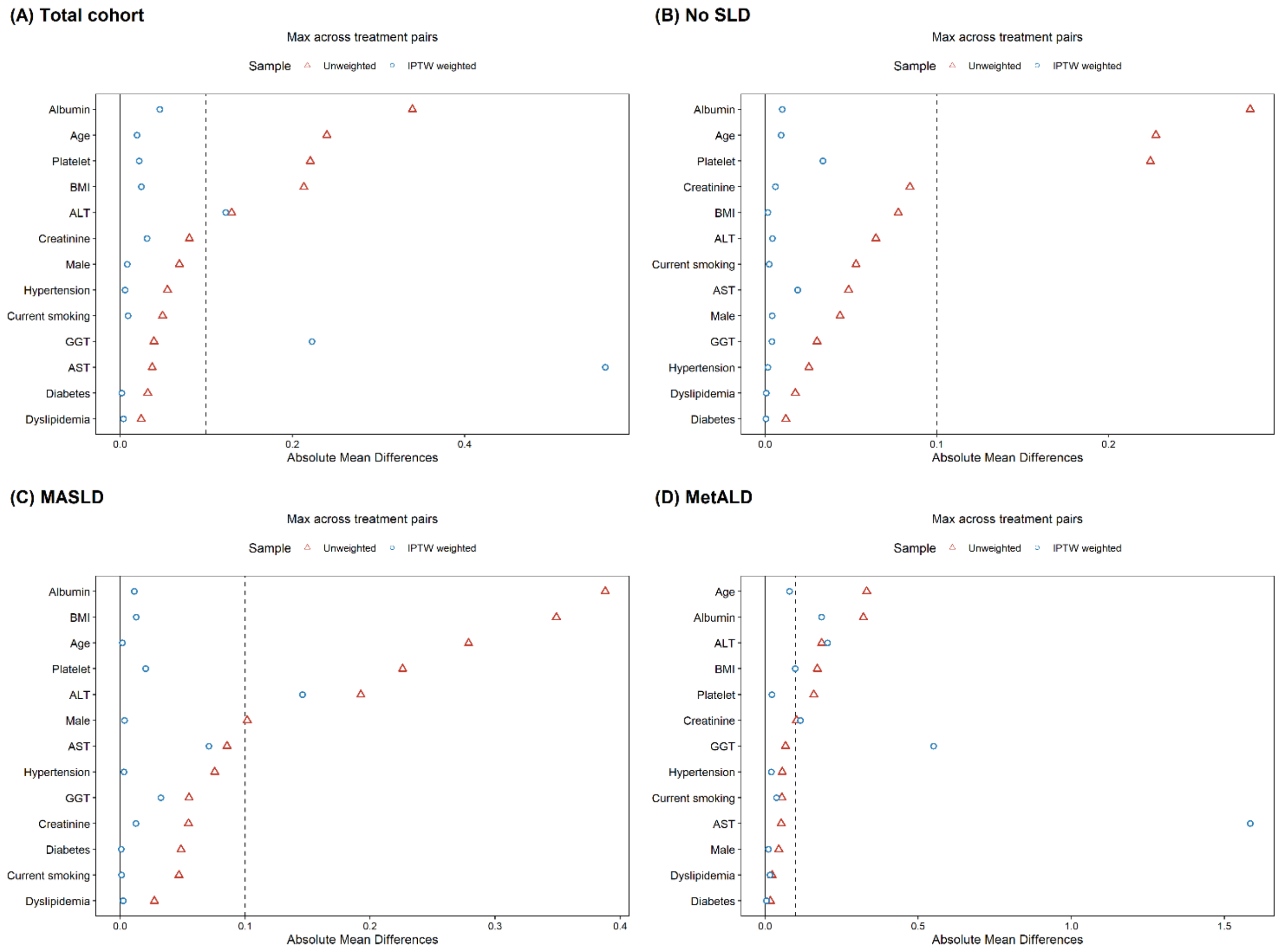

Supplementary Fig. 9. Covariate balance after IPTW. Standardized mean differences before and after IPTW across RDW quartiles in the Total (A), No SLD (B), MASLD (C), and MetALD (D) cohorts. The dashed line indicates the conventional balance threshold of 0.10. IPTW, inverse probability of treatment weighting; RDW, red cell distribution width; SMD, standardized mean difference; SLD, steatotic liver disease; MASLD, metabolic dysfunction-associated steatotic liver disease; MetALD, MASLD with increased alcohol intake.

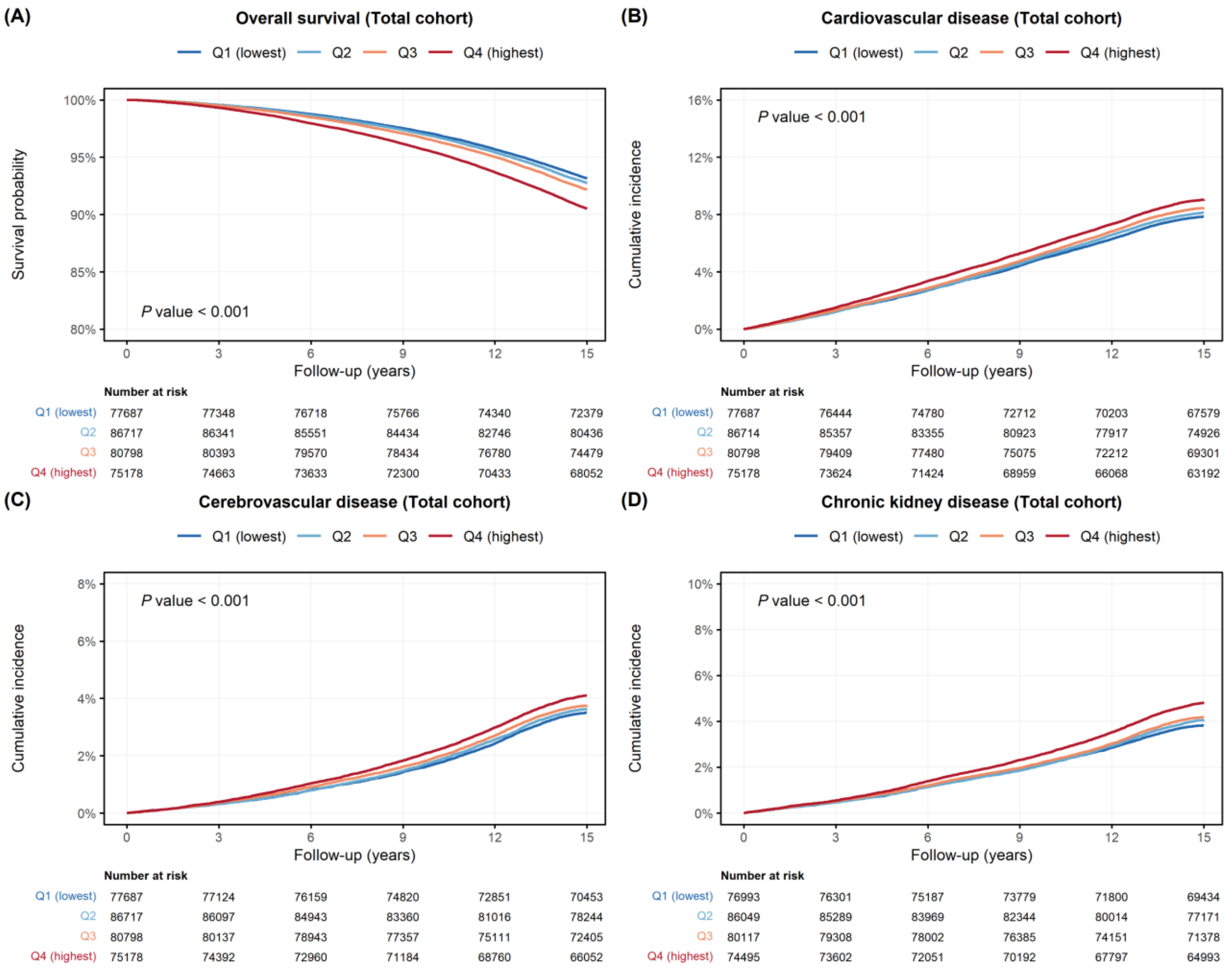

Supplementary Fig. 10. IPTW-weighted event curves in the Total cohort. IPTW-weighted Kaplan-Meier and cumulative-incidence curves for all-cause mortality (A), cardiovascular disease (B), cerebrovascular disease (C), and chronic kidney disease (D) according to RDW quartile in the Total cohort. IPTW, inverse probability of treatment weighting; RDW, red cell distribution width.

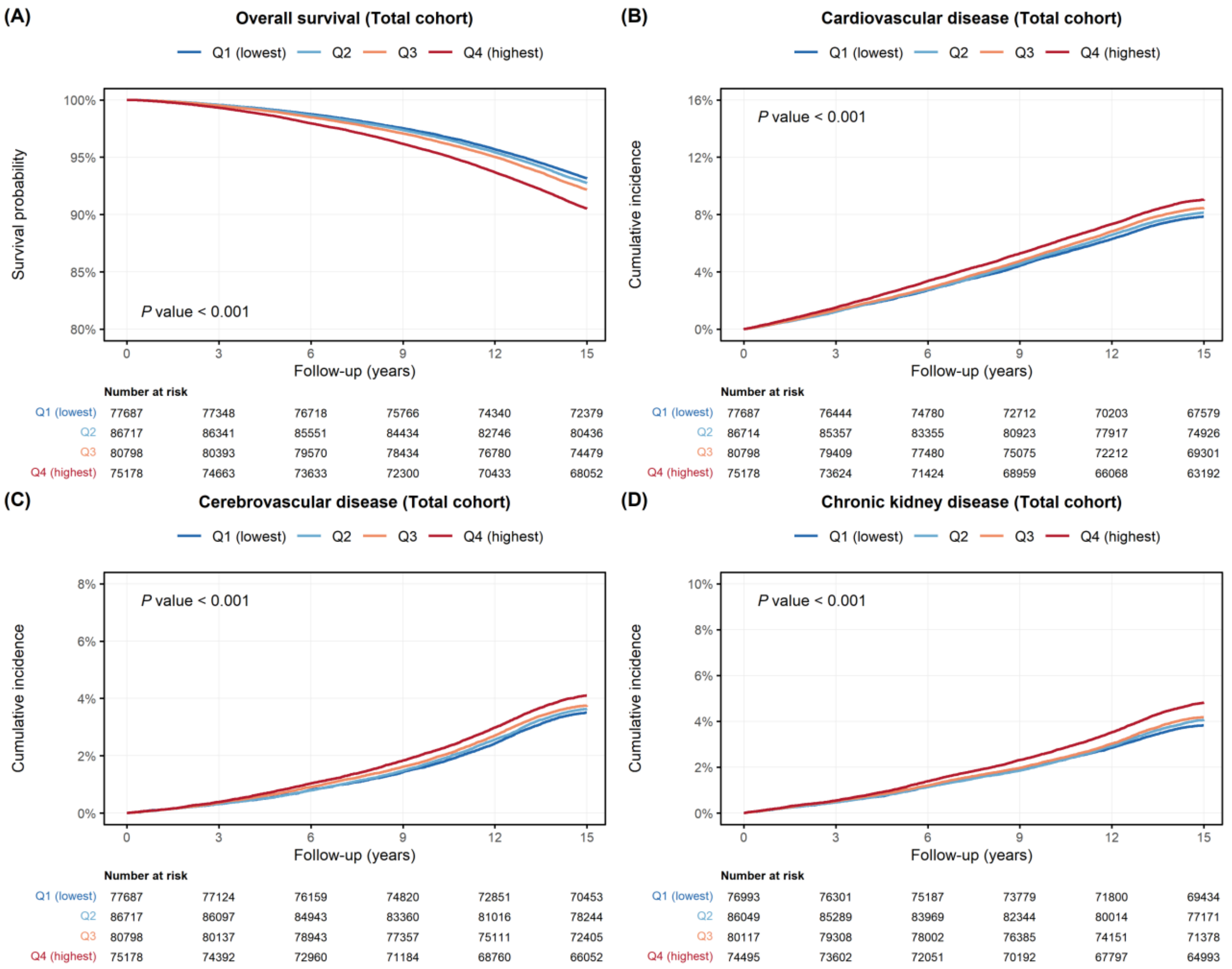

Supplementary Fig. 11. IPTW-weighted event curves in the No SLD cohort. IPTW-weighted Kaplan-Meier and cumulative-incidence curves for all-cause mortality (A), cardiovascular disease (B), cerebrovascular disease (C), and chronic kidney disease (D) according to RDW quartile in the No SLD cohort. IPTW, inverse probability of treatment weighting; RDW, red cell distribution width; SLD, steatotic liver disease.

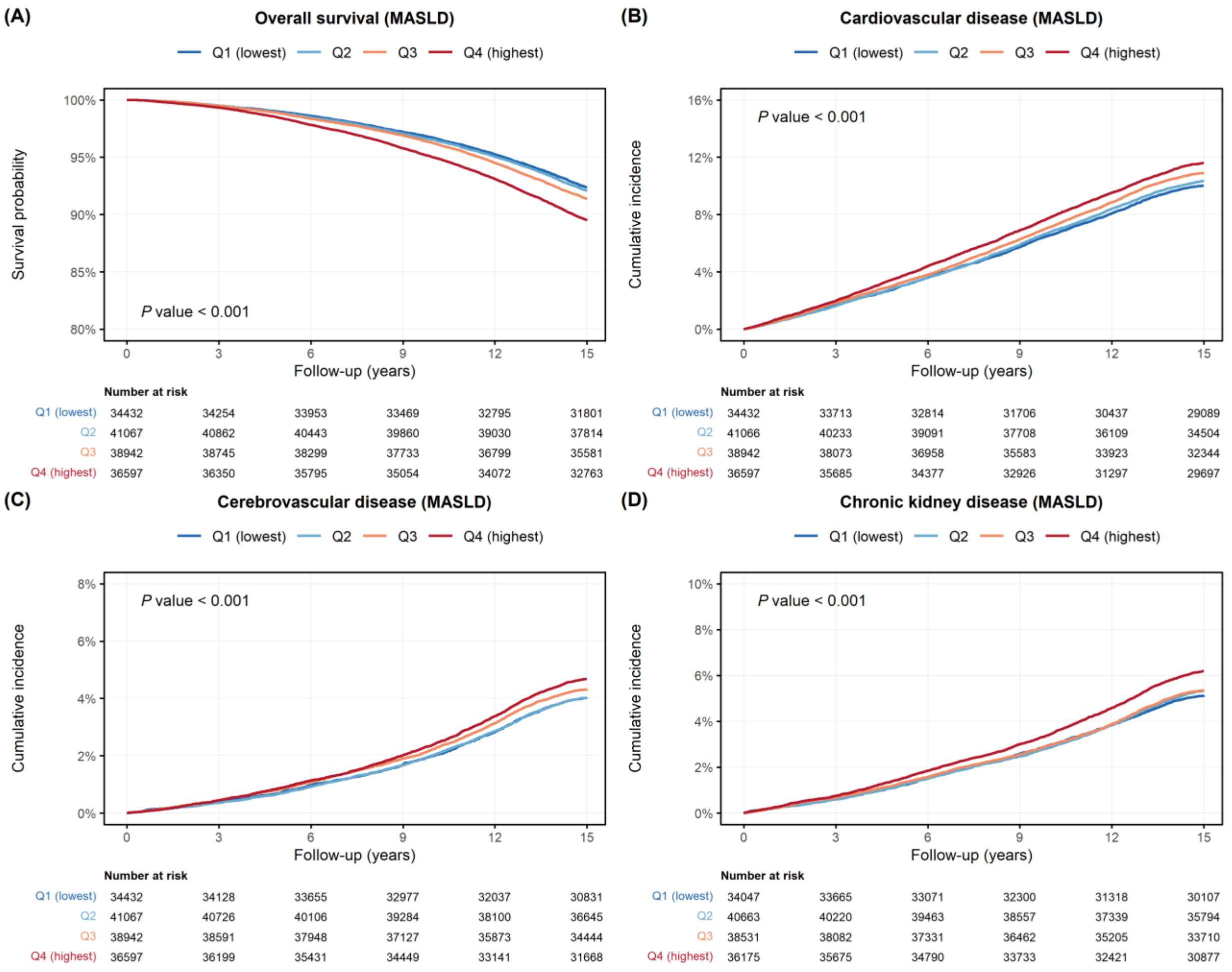

Supplementary Fig. 12. IPTW-weighted event curves in the MASLD cohort. IPTW-weighted Kaplan-Meier and cumulative-incidence curves for all-cause mortality, cardiovascular disease, cerebrovascular disease, and chronic kidney disease according to RDW quartile in the MASLD cohort. IPTW, inverse probability of treatment weighting; RDW, red cell distribution width; MASLD, metabolic dysfunction-associated steatotic liver disease.

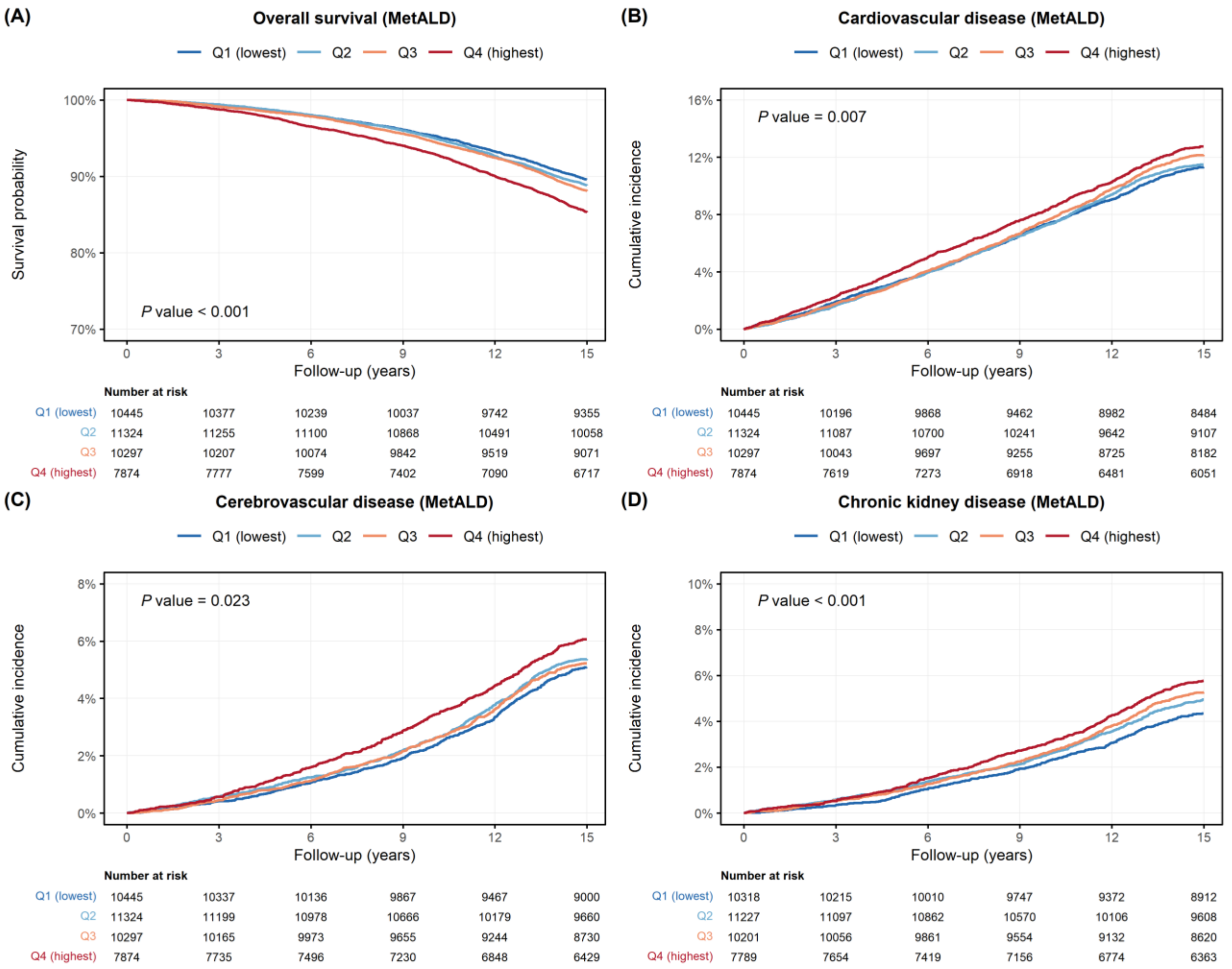

Supplementary Fig. 13. IPTW-weighted event curves in the MetALD cohort. IPTW-weighted Kaplan-Meier and cumulative-incidence curves for all-cause mortality, cardiovascular disease, cerebrovascular disease, and chronic kidney disease according to RDW quartile in the MetALD cohort. IPTW, inverse probability of treatment weighting; RDW, red cell distribution width; MetALD, MASLD with increased alcohol intake.

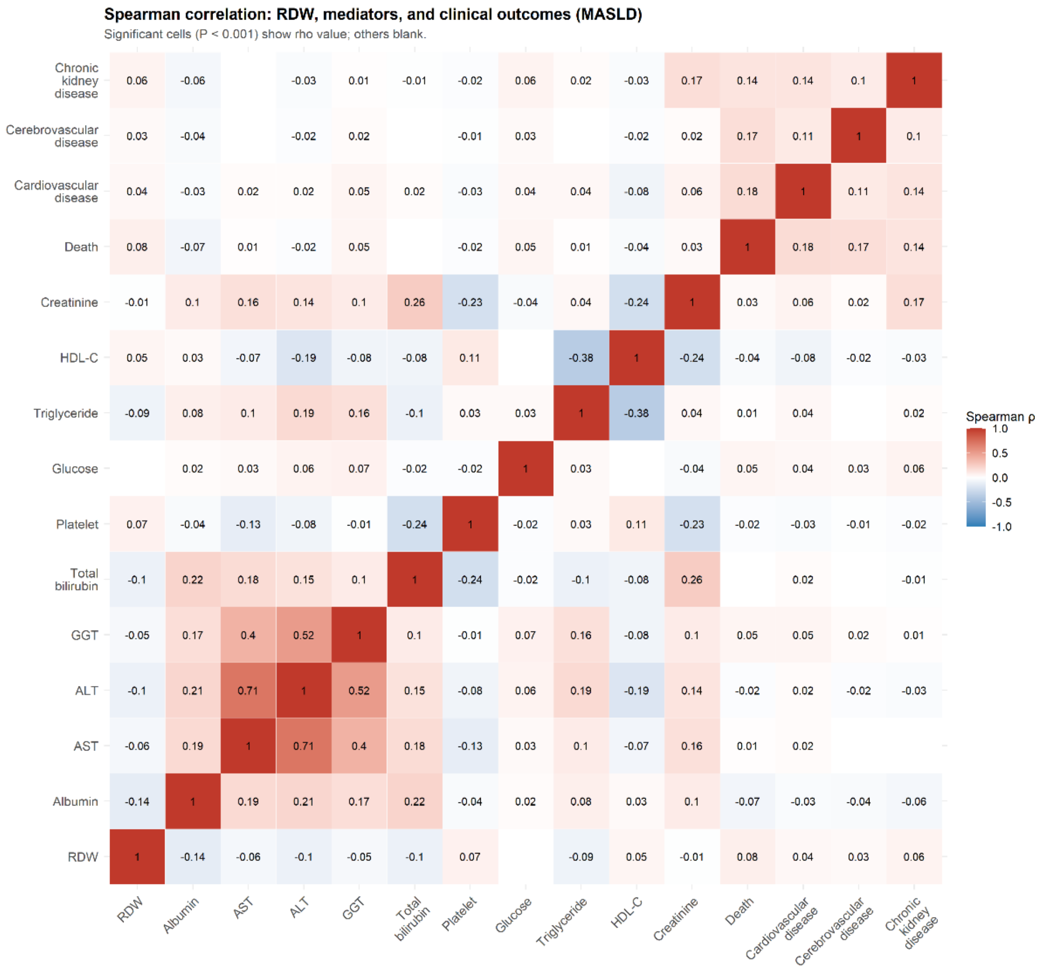

Supplementary Fig. 14. Correlation structure among RDW, candidate biomarkers, and outcomes. Spearman correlation heatmap of RDW, ten candidate biomarkers, and four clinical outcome indicators in the MASLD cohort. Cells display correlation coefficients for statistically significant associations. RDW, red cell distribution width; MASLD, metabolic dysfunction-associated steatotic liver disease; AST, aspartate aminotransferase; ALT, alanine aminotransferase; GGT, gamma-glutamyl transferase; HDL-C, high-density lipoprotein cholesterol.

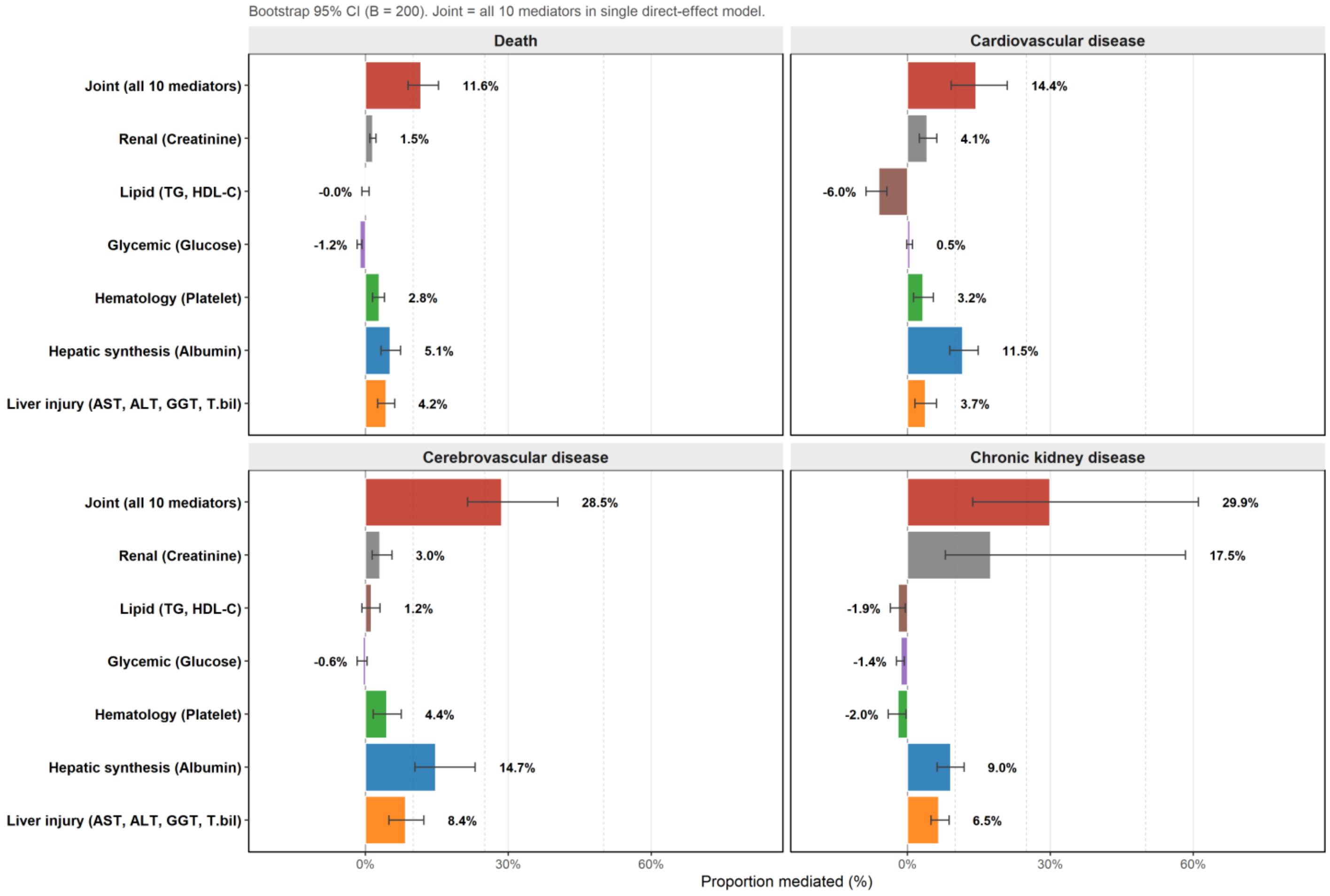

Supplementary Fig. 15. Grouped mediation analysis in MASLD. Grouped mediation estimates for the RDW-outcome associations in the MASLD cohort. Biomarkers were grouped as joint, renal, lipid, glycemic, hematologic, hepatic synthesis, and liver injury categories. Bars indicate proportions mediated with 95% CIs. RDW, red cell distribution width; MASLD, metabolic dysfunction-associated steatotic liver disease; AST, aspartate aminotransferase; ALT, alanine aminotransferase; GGT, gamma-glutamyl transferase; HDL-C, high-density lipoprotein cholesterol.

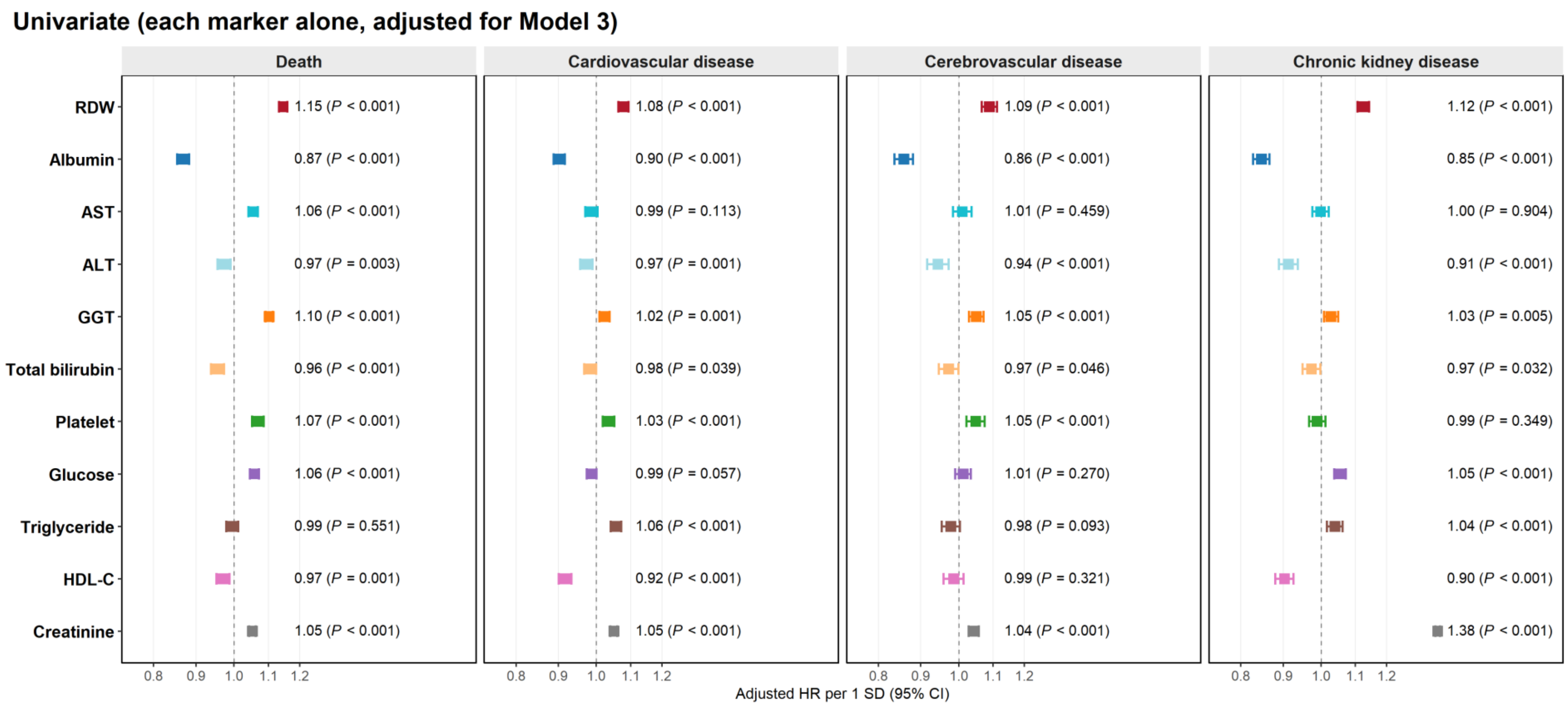

Supplementary Fig. 16. Single-marker Cox analyses in MASLD. HRs for each candidate biomarker entered individually into Cox models for all-cause mortality, cardiovascular disease, cerebrovascular disease, and chronic kidney disease in the MASLD cohort. Estimates are shown per one standard deviation increase. MASLD, metabolic dysfunction-associated steatotic liver disease; RDW, red cell distribution width; HR, hazard ratio; CI, confidence interval; SD, standard deviation.

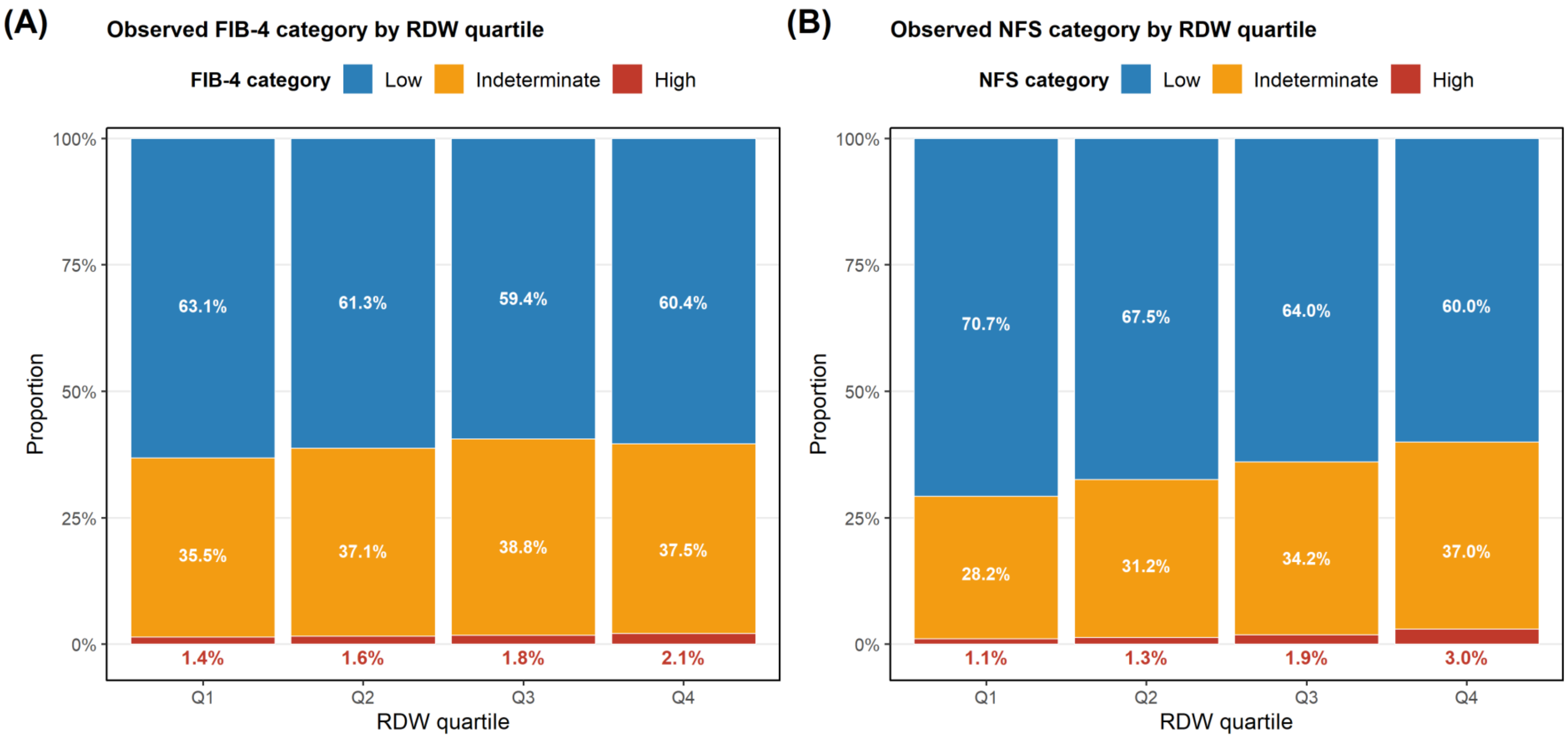

Supplementary Fig. 17. Distribution of fibrosis-score categories by RDW quartile. Observed proportions of FIB-4 (A) and NFS (B) categories across RDW quartiles in the MASLD cohort. Bars show low, indeterminate, and high fibrosis-score categories. RDW, red cell distribution width; MASLD, metabolic dysfunction-associated steatotic liver disease; FIB-4, fibrosis-4 index; NFS, NAFLD fibrosis score.

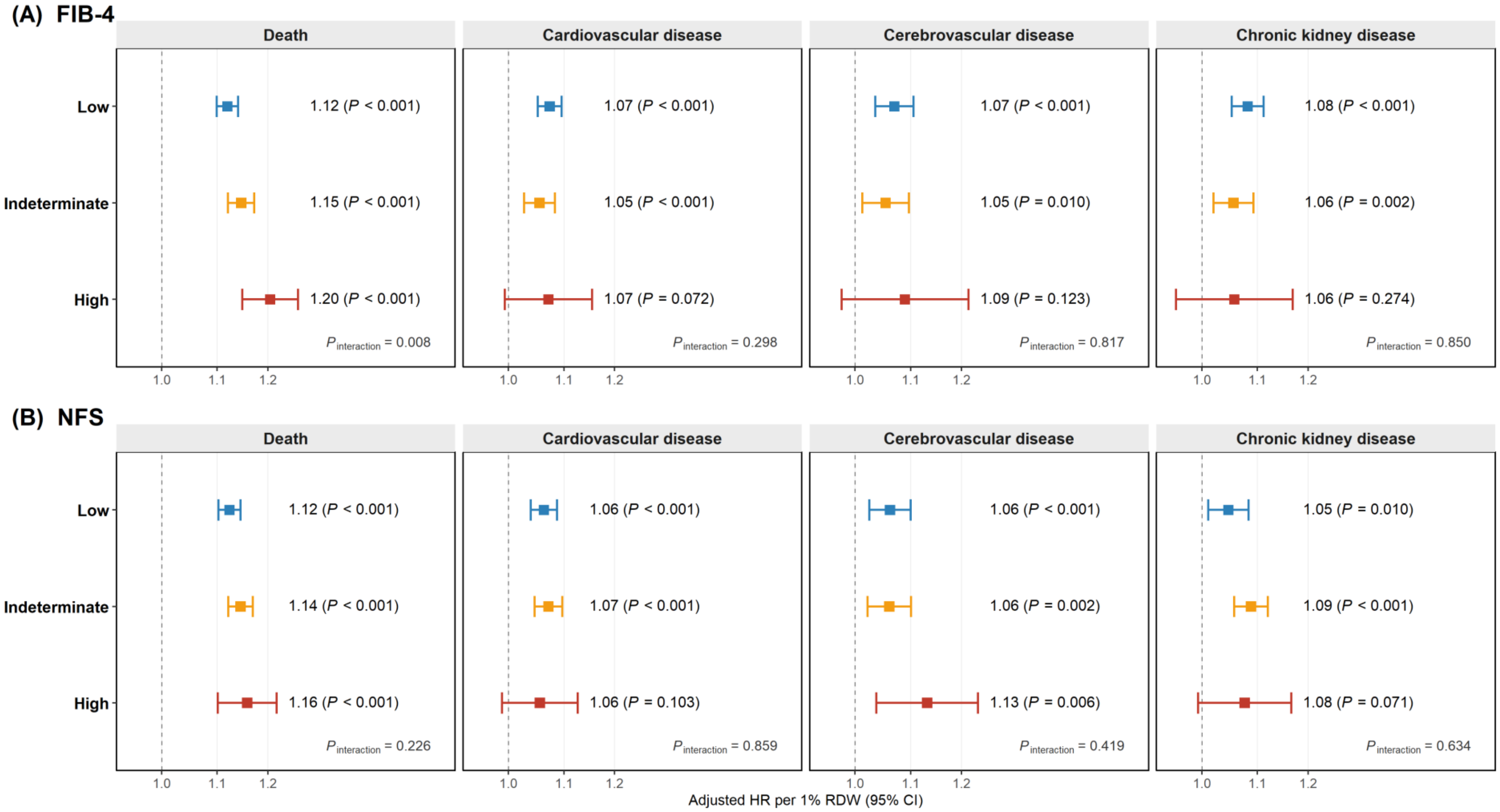

Supplementary Fig. 18. RDW-outcome associations stratified by fibrosis-score category. Model 4-adjusted hazard ratios per 1% increase in RDW for clinical outcomes stratified by FIB-4 (A) and NFS (B) categories in the MASLD cohort. Dots indicate HRs and bars indicate 95% CIs. RDW, red cell distribution width; MASLD, metabolic dysfunction-associated steatotic liver disease; FIB-4, fibrosis-4 index; NFS, NAFLD fibrosis score; HR, hazard ratio; CI, confidence interval.

Supplementary Tables

- **Supplementary Table 1.** Baseline characteristics of the total cohort by RDW quartile.
- **Supplementary Table 2.** Baseline characteristics of the no SLD cohort by RDW quartile.
- **Supplementary Table 3.** Baseline characteristics of the MetALD cohort by RDW quartile.
- **Supplementary Table 4.** Cox proportional hazards models for RDW quartiles and clinical outcomes in the total cohort.
- **Supplementary Table 5.** Cox proportional hazards models for RDW quartiles and clinical outcomes in the no SLD cohort.
- **Supplementary Table 6.** Cox proportional hazards models for RDW quartiles and clinical outcomes in the MetALD cohort.
- **Supplementary Table 7.** Baseline characteristics of the total cohort by RDW quartile after IPTW weighting.
- **Supplementary Table 8.** Baseline characteristics of the no SLD cohort by RDW quartile after IPTW weighting.
- **Supplementary Table 9.** Baseline characteristics of the MASLD cohort by RDW quartile after IPTW weighting.
- **Supplementary Table 10.** Baseline characteristics of the MetALD cohort by RDW quartile after IPTW weighting.
- **Supplementary Table 11.** IPTW-weighted Cox regression of RDW quartile on clinical outcomes.
- **Supplementary Table 12.** Mediation and joint Cox marker comparison in the MASLD cohort.

- **Supplementary Table 13.** Cox regression of RDW per 1% across clinical outcomes, stratified by FIB-4 category in the MASLD cohort.
- **Supplementary Table 14.** Cox regression of RDW per 1% across clinical outcomes, stratified by NFS category in the MASLD cohort.

Supplementary Table 1. Baseline characteristics of the total cohort by RDW quartile.

Characteristic	Overall N = 352,623[1]	Q1 N = 86,017[1]	Q2 N = 95,261[1]	Q3 N = 88,487[1]	Q4 N = 82,858[1]	*P* value[2]
**Age, years**	55.8 ± 8.1	54.6 ± 8.1	55.7 ± 8.1	56.5 ± 8.0	56.5 ± 8.1	< 0.001
**Male**	159,759 (45.3%)	38,245 (44.5%)	45,622 (47.9%)	41,966 (47.4%)	33,926 (40.9%)	< 0.001
**BMI (kg/m²)**	27.0 ± 4.6	26.5 ± 4.1	26.9 ± 4.3	27.1 ± 4.6	27.5 ± 5.2	< 0.001
**Waist circumference (cm)**	89.0 ± 13.2	87.8 ± 12.6	89.0 ± 12.9	89.6 ± 13.2	89.8 ± 14.1	< 0.001
**Smoking status**						< 0.001
Never	199,344 (56.5%)	49,515 (57.6%)	54,084 (56.8%)	49,662 (56.1%)	46,083 (55.6%)	
Previous	117,639 (33.4%)	29,688 (34.5%)	32,354 (34.0%)	29,401 (33.2%)	26,196 (31.6%)	
Current	35,640 (10.1%)	6,814 (7.9%)	8,823 (9.3%)	9,424 (10.7%)	10,579 (12.8%)	
**Diabetes**	30,040 (8.5%)	6,301 (7.3%)	7,610 (8.0%)	7,408 (8.4%)	8,721 (10.5%)	< 0.001
**Hypertension**	135,020 (38.3%)	30,729 (35.7%)	35,719 (37.5%)	34,511 (39.0%)	34,061 (41.1%)	< 0.001
**Dyslipidemia**	88,605 (25.1%)	20,384 (23.7%)	23,844 (25.0%)	22,723 (25.7%)	21,654 (26.1%)	< 0.001
**Glucose (mg/dL)**	91.5 ± 20.6	91.8 ± 22.3	91.4 ± 20.1	91.2 ± 19.3	91.5 ± 20.5	< 0.001
**Triglyceride (mg/dL)**	150.3 ± 89.2	155.6 ± 94.5	153.1 ± 91.0	149.6 ± 87.0	142.3 ± 82.8	< 0.001
**HDL-C (mg/dL)**	56.9 ± 14.8	56.4 ± 14.4	56.5 ± 14.6	57.1 ± 14.9	57.7 ± 15.3	< 0.001
**Creatinine (mg/dL)**	0.8 ± 0.2	0.8 ± 0.2	0.8 ± 0.2	0.8 ± 0.2	0.8 ± 0.2	< 0.001
**AST (U/L)**	25.9 ± 9.8	26.0 ± 9.4	26.0 ± 9.3	25.8 ± 9.2	25.7 ± 11.2	< 0.001
**ALT (U/L)**	23.1 ± 13.6	23.7 ± 13.8	23.5 ± 13.9	23.0 ± 13.4	21.9 ± 13.1	< 0.001
**GGT (U/L)**	35.3 ± 36.8	35.9 ± 36.5	35.5 ± 35.6	35.2 ± 36.2	34.5 ± 39.0	< 0.001
**Albumin, (g/dL)**	4.5 ± 0.3	4.6 ± 0.3	4.5 ± 0.3	4.5 ± 0.3	4.5 ± 0.3	< 0.001
**Platelet (10^9/L)**	253.7 ± 59.0	249.8 ± 54.9	250.9 ± 55.7	252.1 ± 57.1	262.8 ± 67.4	< 0.001
**Total bilirubin (mg/dL)**	0.5 ± 0.3	0.6 ± 0.3	0.5 ± 0.3	0.5 ± 0.3	0.5 ± 0.3	< 0.001
**RDW (%)**	13.5 ± 0.9	12.6 ± 0.3	13.1 ± 0.1	13.6 ± 0.1	14.7 ± 1.1	< 0.001

[1]Mean ± SD; n (%)
[2]One-way analysis of means (not assuming equal variances); Pearson's Chi-squared test
Values are mean ± SD for continuous variables and n (%) for categorical variables. *P* values are from one-way ANOVA for continuous variables and chi-squared test for categorical variables across RDW quartiles. RDW quartiles were defined in the analytic cohort. RDW, red cell distribution width; BMI, body mass index; AST, aspartate aminotransferase; ALT, alanine aminotransferase; GGT, gamma-glutamyl transferase; HDL-C, high-density lipoprotein cholesterol.

Supplementary Table 2. Baseline characteristics of the no SLD cohort by RDW quartile.

Characteristic	Overall<br>N = 143,178[1]	Q1<br>N = 36,452[1]	Q2<br>N = 37,953[1]	Q3<br>N = 34,861[1]	Q4<br>N = 33,912[1]	*P* value[2]
**Age, years**	54.6 ± 8.2	53.5 ± 8.1	54.6 ± 8.1	55.4 ± 8.1	55.1 ± 8.3	< 0.001
**Male**	32,700 (22.8%)	7,474 (20.5%)	9,085 (23.9%)	8,673 (24.9%)	7,468 (22.0%)	< 0.001
**BMI (kg/m²)**	23.5 ± 2.3	23.4 ± 2.3	23.5 ± 2.3	23.6 ± 2.3	23.6 ± 2.4	< 0.001
**Waist circumference (cm)**	77.6 ± 7.1	77.2 ± 7.0	77.6 ± 7.1	77.8 ± 7.1	77.7 ± 7.1	< 0.001
**Smoking status**						< 0.001
Never	88,908 (62.1%)	22,953 (63.0%)	23,777 (62.6%)	21,498 (61.7%)	20,680 (61.0%)	
Previous	40,944 (28.6%)	10,966 (30.1%)	10,980 (28.9%)	9,883 (28.3%)	9,115 (26.9%)	
Current	13,326 (9.3%)	2,533 (6.9%)	3,196 (8.4%)	3,480 (10.0%)	4,117 (12.1%)	
**Diabetes**	3,941 (2.8%)	837 (2.3%)	974 (2.6%)	960 (2.8%)	1,170 (3.5%)	< 0.001
**Hypertension**	34,324 (24.0%)	8,294 (22.8%)	8,921 (23.5%)	8,566 (24.6%)	8,543 (25.2%)	< 0.001
**Dyslipidemia**	21,270 (14.9%)	4,999 (13.7%)	5,673 (14.9%)	5,343 (15.3%)	5,255 (15.5%)	< 0.001
**Glucose (mg/dL)**	88.6 ± 15.1	88.7 ± 15.5	88.5 ± 14.7	88.7 ± 15.3	88.3 ± 14.9	0.001
**Triglyceride (mg/dL)**	99.0 ± 39.7	100.9 ± 40.9	99.7 ± 39.9	98.6 ± 39.2	96.8 ± 38.7	< 0.001
**HDL-C (mg/dL)**	64.7 ± 14.5	64.0 ± 14.1	64.5 ± 14.3	65.3 ± 14.6	65.2 ± 15.1	< 0.001
**Creatinine (mg/dL)**	0.8 ± 0.2	0.8 ± 0.1	0.8 ± 0.1	0.8 ± 0.1	0.8 ± 0.2	< 0.001
**AST (U/L)**	23.8 ± 7.4	23.6 ± 6.9	23.7 ± 7.1	23.9 ± 7.2	23.9 ± 8.6	< 0.001
**ALT (U/L)**	17.4 ± 7.6	17.4 ± 7.2	17.5 ± 7.9	17.5 ± 7.4	17.0 ± 8.1	< 0.001
**GGT (U/L)**	21.4 ± 12.5	21.4 ± 12.2	21.5 ± 12.4	21.5 ± 12.6	21.2 ± 13.1	0.003
**Albumin, (g/dL)**	4.5 ± 0.3	4.6 ± 0.3	4.5 ± 0.3	4.5 ± 0.3	4.5 ± 0.3	< 0.001
**Platelet (10^9/L)**	254.8 ± 58.4	250.8 ± 54.2	251.9 ± 55.3	253.4 ± 56.9	263.9 ± 66.2	< 0.001
**Total bilirubin (mg/dL)**	0.5 ± 0.3	0.6 ± 0.3	0.5 ± 0.3	0.5 ± 0.3	0.5 ± 0.2	< 0.001
**RDW (%)**	13.5 ± 1.0	12.5 ± 0.3	13.1 ± 0.1	13.6 ± 0.1	14.7 ± 1.2	< 0.001

[1]Mean ± SD; n (%)
[2]One-way analysis of means (not assuming equal variances); Pearson's Chi-squared test
Values are mean ± SD for continuous variables and n (%) for categorical variables. *P* values are from one-way ANOVA for continuous variables and chi-squared test for categorical variables across RDW quartiles. RDW quartiles were defined in the analytic cohort. RDW, red cell distribution width; BMI, body mass index; AST, aspartate aminotransferase; ALT, alanine aminotransferase; GGT, gamma-glutamyl transferase; HDL-C, high-density lipoprotein cholesterol.

Supplementary Table 3. Baseline characteristics of the MetALD cohort by RDW quartile.

Characteristic	Overall N = 43,745[1]	Q1 N = 11,502[1]	Q2 N = 12,370[1]	Q3 N = 11,215[1]	Q4 N = 8,658[1]	*P* value[2]
**Age, years**	58.3 ± 7.5	57.0 ± 7.7	58.1 ± 7.5	58.9 ± 7.2	59.4 ± 7.1	< 0.001
**Male**	30,893 (70.6%)	8,000 (69.6%)	8,963 (72.5%)	8,053 (71.8%)	5,877 (67.9%)	< 0.001
**BMI (kg/m²)**	28.4 ± 3.6	28.2 ± 3.4	28.4 ± 3.5	28.5 ± 3.6	28.8 ± 4.0	< 0.001
**Waist circumference (cm)**	96.6 ± 9.7	95.8 ± 9.5	96.7 ± 9.6	96.7 ± 9.7	97.4 ± 10.3	< 0.001
**Smoking status**						< 0.001
Never	17,280 (39.5%)	4,694 (40.8%)	4,873 (39.4%)	4,451 (39.7%)	3,262 (37.7%)	
Previous	20,487 (46.8%)	5,459 (47.5%)	5,921 (47.9%)	5,195 (46.3%)	3,912 (45.2%)	
Current	5,978 (13.7%)	1,349 (11.7%)	1,576 (12.7%)	1,569 (14.0%)	1,484 (17.1%)	
**Diabetes**	4,363 (10.0%)	1,133 (9.9%)	1,192 (9.6%)	1,068 (9.5%)	970 (11.2%)	< 0.001
**Hypertension**	22,340 (51.1%)	5,641 (49.0%)	6,294 (50.9%)	5,706 (50.9%)	4,699 (54.3%)	< 0.001
**Dyslipidemia**	15,007 (34.3%)	3,872 (33.7%)	4,230 (34.2%)	3,817 (34.0%)	3,088 (35.7%)	0.022
**Glucose (mg/dL)**	93.6 ± 21.2	94.6 ± 24.4	93.4 ± 20.0	93.2 ± 19.7	93.2 ± 20.2	< 0.001
**Triglyceride (mg/dL)**	182.4 ± 97.3	194.9 ± 104.0	184.0 ± 98.0	176.4 ± 91.4	171.3 ± 92.6	< 0.001
**HDL-C (mg/dL)**	55.7 ± 13.5	54.6 ± 13.0	55.2 ± 13.0	56.2 ± 13.5	57.5 ± 14.6	< 0.001
**Creatinine (mg/dL)**	0.9 ± 0.2	0.8 ± 0.2	0.9 ± 0.2	0.9 ± 0.2	0.9 ± 0.2	< 0.001
**AST (U/L)**	28.5 ± 12.7	28.9 ± 13.2	28.5 ± 11.9	28.1 ± 11.4	28.5 ± 14.5	< 0.001
**ALT (U/L)**	27.8 ± 16.2	29.2 ± 16.4	28.2 ± 16.6	27.2 ± 15.8	26.2 ± 15.7	< 0.001
**GGT (U/L)**	54.6 ± 56.9	56.9 ± 57.8	53.9 ± 53.4	52.8 ± 54.6	54.9 ± 63.2	< 0.001
**Albumin, (g/dL)**	4.5 ± 0.3	4.6 ± 0.3	4.6 ± 0.3	4.5 ± 0.3	4.5 ± 0.3	< 0.001
**Platelet (10⁹/L)**	247.8 ± 57.2	245.2 ± 54.7	246.3 ± 55.4	247.2 ± 55.7	254.4 ± 64.3	< 0.001
**Total bilirubin (mg/dL)**	0.6 ± 0.2	0.6 ± 0.2	0.6 ± 0.3	0.6 ± 0.2	0.5 ± 0.3	< 0.001
**RDW (%)**	13.4 ± 0.8	12.6 ± 0.3	13.1 ± 0.1	13.6 ± 0.1	14.5 ± 0.9	< 0.001

[1]Mean ± SD; n (%)
[2]One-way analysis of means (not assuming equal variances); Pearson's Chi-squared test
Values are mean ± SD for continuous variables and n (%) for categorical variables. *P* values are from one-way ANOVA for continuous variables and chi-squared test for categorical variables across RDW quartiles. RDW quartiles were defined in the analytic cohort. RDW, red cell distribution width; BMI, body mass index; AST, aspartate aminotransferase; ALT, alanine aminotransferase; GGT, gamma-glutamyl transferase; HDL-C, high-density lipoprotein cholesterol.

Supplementary Table 4. Cox proportional hazards models for RDW quartiles and clinical outcomes in the total cohort.

Outcome	Form	Model 1	Model 2	Model 3	Model 4
**Death**	Q1 (ref)	1.00 (ref)	1.00 (ref)	1.00 (ref)	1.00 (ref)
	Q2 vs Q1	1.23 (1.18–1.27) *P* < 0.001	1.09 (1.05–1.13) *P* < 0.001	1.07 (1.04–1.11) *P* < 0.001	1.07 (1.03–1.11) *P* < 0.001
	Q3 vs Q1	1.45 (1.40–1.50) *P* < 0.001	1.20 (1.16–1.24) *P* < 0.001	1.16 (1.12–1.20) *P* < 0.001	1.14 (1.10–1.18) *P* < 0.001
	Q4 vs Q1	1.89 (1.82–1.95) *P* < 0.001	1.58 (1.53–1.63) *P* < 0.001	1.45 (1.40–1.50) *P* < 0.001	1.38 (1.33–1.43) *P* < 0.001
	P-trend	*P* < 0.001	*P* < 0.001	*P* < 0.001	*P* < 0.001
**Cardiovascular disease**	Q1 (ref)	1.00 (ref)	1.00 (ref)	1.00 (ref)	1.00 (ref)
	Q2 vs Q1	1.15 (1.11–1.19) *P* < 0.001	1.04 (1.00–1.07) *P* = 0.026	1.04 (1.00–1.07) *P* = 0.034	1.03 (0.99–1.07) *P* = 0.112
	Q3 vs Q1	1.27 (1.23–1.31) *P* < 0.001	1.09 (1.06–1.13) *P* < 0.001	1.09 (1.05–1.13) *P* < 0.001	1.07 (1.04–1.11) *P* < 0.001
	Q4 vs Q1	1.42 (1.37–1.47) *P* < 0.001	1.26 (1.22–1.31) *P* < 0.001	1.19 (1.15–1.23) *P* < 0.001	1.15 (1.11–1.19) *P* < 0.001
	P-trend	*P* < 0.001	*P* < 0.001	*P* < 0.001	*P* < 0.001
**Cerebrovascular disease**	Q1 (ref)	1.00 (ref)	1.00 (ref)	1.00 (ref)	1.00 (ref)
	Q2 vs Q1	1.16 (1.10–1.22) *P* < 0.001	1.04 (0.98–1.09) *P* = 0.171	1.04 (0.99–1.09) *P* = 0.162	1.03 (0.98–1.09) *P* = 0.268
	Q3 vs Q1	1.31 (1.24–1.38) *P* < 0.001	1.09 (1.03–1.14) *P* = 0.002	1.08 (1.03–1.14) *P* = 0.002	1.06 (1.01–1.12) *P* = 0.025
	Q4 vs Q1	1.54 (1.46–1.62) *P* < 0.001	1.29 (1.23–1.36) *P* < 0.001	1.23 (1.17–1.29) *P* < 0.001	1.16 (1.10–1.22) *P* < 0.001
	P-trend	*P* < 0.001	*P* < 0.001	*P* < 0.001	*P* < 0.001
**Chronic kidney disease**	Q1 (ref)	1.00 (ref)	1.00 (ref)	1.00 (ref)	1.00 (ref)
	Q2 vs Q1	1.20 (1.14–1.27) *P* < 0.001	1.08 (1.03–1.14) *P* = 0.002	1.07 (1.02–1.13) *P* = 0.006	1.05 (1.00–1.11) *P* = 0.065
	Q3 vs Q1	1.42 (1.36–1.50) *P* < 0.001	1.19 (1.13–1.25) *P* < 0.001	1.16 (1.11–1.22) *P* < 0.001	1.11 (1.06–1.17) *P* < 0.001
	Q4 vs Q1	1.89 (1.80–1.98) *P* < 0.001	1.58 (1.50–1.65) *P* < 0.001	1.43 (1.36–1.50) *P* < 0.001	1.25 (1.19–1.32) *P* < 0.001
	P-trend	*P* < 0.001	*P* < 0.001	*P* < 0.001	*P* < 0.001

Hazard ratios (HR) with 95% confidence intervals (CI) from Cox proportional hazards models comparing RDW quartiles (Q1 = reference). Model 1: crude. Model 2: adjusted for age and sex. Model 3: Model 2 + BMI, diabetes, hypertension, dyslipidemia, and smoking status. Model 4: Model 3 + AST, ALT, GGT, platelet count, albumin, and creatinine. Previous events at baseline were excluded from each outcome analysis. *P*-trend was tested by modeling RDW quartile as an ordinal variable. RDW, red cell distribution width; AST, aspartate aminotransferase; ALT, alanine aminotransferase; GGT, gamma-glutamyl transferase.

Supplementary Table 5. Cox proportional hazards models for RDW quartiles and clinical outcomes in the no SLD cohort.

Outcome	Form	Model 1	Model 2	Model 3	Model 4
**Death**	Q1 (ref)	1.00 (ref)	1.00 (ref)	1.00 (ref)	1.00 (ref)
	Q2 vs Q1	1.24 (1.16–1.32) *P* < 0.001	1.09 (1.03–1.16) *P* = 0.006	1.07 (1.00–1.14) *P* = 0.037	1.06 (0.99–1.13) *P* = 0.086
	Q3 vs Q1	1.45 (1.36–1.54) *P* < 0.001	1.17 (1.10–1.25) *P* < 0.001	1.13 (1.06–1.20) *P* < 0.001	1.11 (1.04–1.19) *P* = 0.001
	Q4 vs Q1	1.82 (1.72–1.93) *P* < 0.001	1.50 (1.42–1.60) *P* < 0.001	1.36 (1.28–1.44) *P* < 0.001	1.29 (1.21–1.38) *P* < 0.001
	P-trend	*P* < 0.001	*P* < 0.001	*P* < 0.001	*P* < 0.001
**Cardiovascular disease**	Q1 (ref)	1.00 (ref)	1.00 (ref)	1.00 (ref)	1.00 (ref)
	Q2 vs Q1	1.18 (1.10–1.27) *P* < 0.001	1.05 (0.98–1.13) *P* = 0.185	1.05 (0.98–1.12) *P* = 0.205	1.03 (0.95–1.11) *P* = 0.468
	Q3 vs Q1	1.26 (1.17–1.35) *P* < 0.001	1.04 (0.97–1.11) *P* = 0.313	1.04 (0.97–1.11) *P* = 0.315	1.02 (0.94–1.10) *P* = 0.642
	Q4 vs Q1	1.45 (1.35–1.55) *P* < 0.001	1.23 (1.14–1.32) *P* < 0.001	1.15 (1.07–1.24) *P* < 0.001	1.09 (1.02–1.18) *P* = 0.019
	P-trend	*P* < 0.001	*P* < 0.001	*P* < 0.001	*P* = 0.027
**Cerebrovascular disease**	Q1 (ref)	1.00 (ref)	1.00 (ref)	1.00 (ref)	1.00 (ref)
	Q2 vs Q1	1.21 (1.10–1.33) *P* < 0.001	1.06 (0.97–1.17) *P* = 0.204	1.06 (0.97–1.17) *P* = 0.196	1.06 (0.96–1.17) *P* = 0.242
	Q3 vs Q1	1.34 (1.22–1.47) *P* < 0.001	1.07 (0.97–1.18) *P* = 0.165	1.07 (0.97–1.18) *P* = 0.163	1.06 (0.96–1.17) *P* = 0.238
	Q4 vs Q1	1.50 (1.37–1.65) *P* < 0.001	1.22 (1.12–1.34) *P* < 0.001	1.17 (1.06–1.28) *P* = 0.001	1.11 (1.00–1.22) *P* = 0.042
	P-trend	*P* < 0.001	*P* < 0.001	*P* = 0.002	*P* = 0.055
**Chronic kidney disease**	Q1 (ref)	1.00 (ref)	1.00 (ref)	1.00 (ref)	1.00 (ref)
	Q2 vs Q1	1.16 (1.05–1.28) *P* = 0.004	1.04 (0.94–1.15) *P* = 0.429	1.05 (0.95–1.16) *P* = 0.354	1.03 (0.93–1.15) *P* = 0.542
	Q3 vs Q1	1.34 (1.21–1.48) *P* < 0.001	1.12 (1.01–1.23) *P* = 0.030	1.11 (1.01–1.23) *P* = 0.038	1.09 (0.98–1.21) *P* = 0.109
	Q4 vs Q1	1.66 (1.51–1.83) *P* < 0.001	1.41 (1.28–1.55) *P* < 0.001	1.35 (1.23–1.49) *P* < 0.001	1.19 (1.08–1.32) *P* < 0.001
	P-trend	*P* < 0.001	*P* < 0.001	*P* < 0.001	*P* < 0.001

Hazard ratios (HR) with 95% confidence intervals (CI) from Cox proportional hazards models comparing RDW quartiles (Q1 = reference). Model 1: crude. Model 2: adjusted for age and sex. Model 3: Model 2 + BMI, diabetes, hypertension, dyslipidemia, and smoking status. Model 4: Model 3 + AST, ALT, GGT, platelet count, albumin, and creatinine. Previous events at baseline were excluded from each outcome analysis. *P*-trend was tested by modeling RDW quartile as an ordinal variable. RDW, red cell distribution width; AST, aspartate aminotransferase; ALT, alanine aminotransferase; GGT, gamma-glutamyl transferase.

Supplementary Table 6. Cox proportional hazards models for RDW quartiles and clinical outcomes in the MetALD cohort.

Outcome	Form	Model 1	Model 2	Model 3	Model 4
**Death**	Q1 (ref)	1.00 (ref)	1.00 (ref)	1.00 (ref)	1.00 (ref)
	Q2 vs Q1	1.25 (1.16–1.35)<br>*P* < 0.001	1.13 (1.05–1.22)<br>*P* = 0.002	1.12 (1.03–1.21)<br>*P* = 0.005	1.11 (1.03–1.21)<br>*P* = 0.009
	Q3 vs Q1	1.43 (1.32–1.54)<br>*P* < 0.001	1.23 (1.14–1.32)<br>*P* < 0.001	1.20 (1.12–1.30)<br>*P* < 0.001	1.19 (1.10–1.29)<br>*P* < 0.001
	Q4 vs Q1	1.97 (1.82–2.12)<br>*P* < 0.001	1.66 (1.54–1.79)<br>*P* < 0.001	1.56 (1.44–1.68)<br>*P* < 0.001	1.50 (1.38–1.62)<br>*P* < 0.001
	P-trend	*P* < 0.001	*P* < 0.001	*P* < 0.001	*P* < 0.001
**Cardiovascular disease**	Q1 (ref)	1.00 (ref)	1.00 (ref)	1.00 (ref)	1.00 (ref)
	Q2 vs Q1	1.11 (1.03–1.20)<br>*P* = 0.006	1.03 (0.95–1.11)<br>*P* = 0.440	1.04 (0.96–1.12)<br>*P* = 0.325	1.02 (0.94–1.10)<br>*P* = 0.693
	Q3 vs Q1	1.20 (1.11–1.30)<br>*P* < 0.001	1.08 (0.99–1.16)<br>*P* = 0.071	1.10 (1.01–1.19)<br>*P* = 0.021	1.09 (1.00–1.18)<br>*P* = 0.044
	Q4 vs Q1	1.36 (1.25–1.48)<br>*P* < 0.001	1.22 (1.12–1.32)<br>*P* < 0.001	1.20 (1.10–1.30)<br>*P* < 0.001	1.16 (1.06–1.26)<br>*P* < 0.001
	P-trend	*P* < 0.001	*P* < 0.001	*P* < 0.001	*P* < 0.001
**Cerebrovascular disease**	Q1 (ref)	1.00 (ref)	1.00 (ref)	1.00 (ref)	1.00 (ref)
	Q2 vs Q1	1.17 (1.04–1.32)<br>*P* = 0.008	1.06 (0.94–1.20)<br>*P* = 0.310	1.08 (0.96–1.21)<br>*P* = 0.206	1.09 (0.96–1.23)<br>*P* = 0.191
	Q3 vs Q1	1.22 (1.08–1.37)<br>*P* = 0.001	1.05 (0.93–1.18)<br>*P* = 0.436	1.07 (0.95–1.21)<br>*P* = 0.272	1.05 (0.93–1.19)<br>*P* = 0.415
	Q4 vs Q1	1.57 (1.39–1.77)<br>*P* < 0.001	1.31 (1.16–1.48)<br>*P* < 0.001	1.29 (1.14–1.45)<br>*P* < 0.001	1.23 (1.08–1.40)<br>*P* = 0.001
	P-trend	*P* < 0.001	*P* < 0.001	*P* < 0.001	*P* = 0.004
**Chronic kidney disease**	Q1 (ref)	1.00 (ref)	1.00 (ref)	1.00 (ref)	1.00 (ref)
	Q2 vs Q1	1.28 (1.13–1.46)<br>*P* < 0.001	1.16 (1.02–1.31)<br>*P* = 0.027	1.15 (1.01–1.31)<br>*P* = 0.030	1.13 (0.99–1.30)<br>*P* = 0.069
	Q3 vs Q1	1.49 (1.31–1.69)<br>*P* < 0.001	1.27 (1.12–1.44)<br>*P* < 0.001	1.29 (1.13–1.46)<br>*P* < 0.001	1.24 (1.08–1.41)<br>*P* = 0.002
	Q4 vs Q1	1.98 (1.74–2.24)<br>*P* < 0.001	1.61 (1.42–1.83)<br>*P* < 0.001	1.54 (1.36–1.75)<br>*P* < 0.001	1.26 (1.09–1.44)<br>*P* = 0.001
	P-trend	*P* < 0.001	*P* < 0.001	*P* < 0.001	*P* < 0.001

Hazard ratios (HR) with 95% confidence intervals (CI) from Cox proportional hazards models comparing RDW quartiles (Q1 = reference). Model 1: crude. Model 2: adjusted for age and sex. Model 3: Model 2 + BMI, diabetes, hypertension, dyslipidemia, and smoking status. Model 4: Model 3 + AST, ALT, GGT, platelet count, albumin, and creatinine. Previous events at baseline were excluded from each outcome analysis. *P*-trend was tested by modeling RDW quartile as an ordinal variable. RDW, red cell distribution width; AST, aspartate aminotransferase; ALT, alanine aminotransferase; GGT, gamma-glutamyl transferase.

Supplementary Table 7. Baseline characteristics of the total cohort by RDW quartile after IPTW weighting.

Variable	IPTW Q1	IPTW Q2	IPTW Q3	IPTW Q4	SMD (Unweighted)	SMD (IPTW)
Number	77687.3	86716.6	80798.0	75177.7	-	-
Age, years	55.8 (8.1)	55.9 (8.1)	55.8 (8.1)	56.0 (8.2)	0.138	0.010
Male sex	35416.4 (45.6)	39648.9 (45.7)	36989.0 (45.8)	33916.8 (45.1)	0.079	0.007
BMI, kg/m²	26.9 (4.3)	27.0 (4.4)	27.0 (4.6)	27.0 (4.8)	0.115	0.008
Waist circumference, cm	89.0 (12.8)	89.1 (13.0)	89.1 (13.3)	89.0 (13.7)	0.083	0.007
Current smoker	7724.0 (9.9)	8824.2 (10.2)	8231.4 (10.2)	7801.5 (10.4)	0.089	0.007
Diabetes	6491.2 (8.4)	7372.8 (8.5)	6906.2 (8.5)	6459.1 (8.6)	0.058	0.005
Hypertension	29619.0 (38.1)	33231.1 (38.3)	31015.1 (38.4)	28916.8 (38.5)	0.062	0.004
Dyslipidemia	19521.8 (25.1)	21867.5 (25.2)	20416.8 (25.3)	18995.2 (25.3)	0.031	0.002
Glucose, mg/dL	92.2 (22.8)	91.5 (20.4)	91.1 (19.5)	91.0 (19.3)	0.015	0.033
Triglyceride, mg/dL	158.1 (93.1)	152.4 (89.3)	148.0 (86.1)	140.8 (82.9)	0.083	0.107
HDL-C, mg/dL	55.9 (14.3)	56.7 (14.5)	57.4 (14.9)	58.0 (15.4)	0.051	0.079
Creatinine, mg/dL	0.8 (0.2)	0.8 (0.2)	0.8 (0.2)	0.8 (0.2)	0.043	0.006
AST, U/L	25.9 (9.3)	25.9 (9.5)	25.9 (9.8)	25.9 (10.2)	0.020	0.004
ALT, U/L	23.2 (12.8)	23.1 (13.4)	23.1 (14.4)	23.0 (14.8)	0.072	0.008
GGT, U/L	35.7 (34.6)	35.4 (36.3)	35.6 (38.8)	35.3 (39.5)	0.021	0.005
Albumin, g/dL	4.5 (0.3)	4.5 (0.3)	4.5 (0.3)	4.5 (0.3)	0.183	0.009
Platelet, ×10⁹/L	253.1 (56.5)	253.4 (57.0)	253.5 (57.9)	253.3 (63.4)	0.110	0.004
RDW, %	12.6 (0.3)	13.1 (0.1)	13.6 (0.1)	14.6 (1.1)	2.781	2.801

IPTW-weighted values are weighted mean (SD) for continuous variables and weighted n (%) for categorical variables. IPTW: inverse probability of treatment weighting with stabilized weights from a multinomial propensity model for RDW quartile conditional on age, sex, BMI, diabetes, hypertension, dyslipidemia, current-smoker status, AST, ALT, GGT, platelet, albumin, and creatinine. Weights were trimmed at the 1st and 99th percentiles to reduce the influence of extreme values. IPTW, inverse probability of treatment weighting; RDW, red cell distribution width; BMI, body mass index; HDL-C, high-density lipoprotein cholesterol; AST, aspartate aminotransferase; ALT, alanine aminotransferase; GGT, gamma-glutamyl transferase; SMD, standardized mean difference.

Supplementary Table 8. Baseline characteristics of the no SLD cohort by RDW quartile after IPTW weighting.

Variable	IPTW Q1	IPTW Q2	IPTW Q3	IPTW Q4	SMD (Unweighted)	SMD (IPTW)
Number	32804.1	34319.3	31555.5	30714.4	-	-
Age, years	54.6 (8.1)	54.7 (8.1)	54.7 (8.2)	54.7 (8.3)	0.125	0.009
Male sex	7460.9 (22.7)	7914.4 (23.1)	7308.1 (23.2)	6980.0 (22.7)	0.059	0.006
BMI, kg/m²	23.5 (2.3)	23.5 (2.3)	23.5 (2.4)	23.5 (2.4)	0.043	0.001
Waist circumference, cm	77.5 (7.0)	77.6 (7.0)	77.5 (7.1)	77.6 (7.2)	0.049	0.004
Current smoker	2929.8 (8.9)	3195.8 (9.3)	2960.3 (9.4)	2908.4 (9.5)	0.100	0.010
Diabetes	880.3 (2.7)	945.1 (2.8)	872.4 (2.8)	861.2 (2.8)	0.038	0.004
Hypertension	7825.1 (23.9)	8232.2 (24.0)	7573.7 (24.0)	7407.6 (24.1)	0.034	0.003
Dyslipidemia	4859.6 (14.8)	5111.8 (14.9)	4713.5 (14.9)	4590.5 (14.9)	0.027	0.002
Glucose, mg/dL	89.0 (16.2)	88.5 (14.8)	88.6 (15.3)	88.3 (14.0)	0.016	0.025
Triglyceride, mg/dL	101.9 (41.1)	99.7 (40.2)	97.9 (39.0)	95.7 (38.1)	0.057	0.086
HDL-C, mg/dL	63.7 (14.1)	64.5 (14.3)	65.3 (14.6)	65.6 (15.1)	0.053	0.074
Creatinine, mg/dL	0.8 (0.1)	0.8 (0.1)	0.8 (0.1)	0.8 (0.2)	0.046	0.005
AST, U/L	23.7 (6.5)	23.7 (7.6)	23.7 (7.1)	23.8 (7.6)	0.027	0.006
ALT, U/L	17.3 (6.9)	17.3 (7.7)	17.3 (7.5)	17.3 (8.2)	0.032	0.001
GGT, U/L	21.5 (11.9)	21.5 (12.5)	21.5 (12.8)	21.5 (13.6)	0.015	0.002
Albumin, g/dL	4.5 (0.3)	4.5 (0.3)	4.5 (0.3)	4.5 (0.3)	0.152	0.007
Platelet, ×10⁹/L	254.1 (55.6)	254.4 (56.7)	254.6 (57.9)	254.5 (62.7)	0.113	0.005
RDW, %	12.5 (0.3)	13.1 (0.1)	13.6 (0.1)	14.7 (1.2)	2.743	2.758

IPTW-weighted values are weighted mean (SD) for continuous variables and weighted n (%) for categorical variables. IPTW: inverse probability of treatment weighting with stabilized weights from a multinomial propensity model for RDW quartile conditional on age, sex, BMI, diabetes, hypertension, dyslipidemia, current-smoker status, AST, ALT, GGT, platelet, albumin, and creatinine. Weights were trimmed at the 1st and 99th percentiles to reduce the influence of extreme values. IPTW, inverse probability of treatment weighting; RDW, red cell distribution width; BMI, body mass index; HDL-C, high-density lipoprotein cholesterol; AST, aspartate aminotransferase; ALT, alanine aminotransferase; GGT, gamma-glutamyl transferase; SMD, standardized mean difference.

Supplementary Table 9. Baseline characteristics of the MASLD cohort by RDW quartile after IPTW weighting.

Variable	IPTW Q1	IPTW Q2	IPTW Q3	IPTW Q4	SMD (Unweighted)	SMD (IPTW)
Number	34431.7	41066.9	38941.7	36597.3	-	-
Age, years	56.2 (8.0)	56.2 (8.0)	56.2 (8.0)	56.4 (8.1)	0.158	0.011
Male sex	20166.7 (58.6)	23978.1 (58.4)	22757.6 (58.4)	21219.7 (58.0)	0.104	0.006
BMI, kg/m²	29.5 (4.1)	29.6 (4.2)	29.6 (4.3)	29.6 (4.4)	0.185	0.013
Waist circumference, cm	96.7 (10.2)	96.9 (10.5)	97.1 (10.5)	97.2 (10.7)	0.139	0.024
Current smoker	3375.5 (9.8)	4085.3 (9.9)	3867.3 (9.9)	3741.6 (10.2)	0.086	0.007
Diabetes	4443.0 (12.9)	5359.3 (13.1)	5109.6 (13.1)	4856.1 (13.3)	0.073	0.006
Hypertension	16206.3 (47.1)	19395.8 (47.2)	18424.6 (47.3)	17394.4 (47.5)	0.083	0.005
Dyslipidemia	10886.2 (31.6)	12973.2 (31.6)	12334.2 (31.7)	11626.4 (31.8)	0.033	0.002
Glucose, mg/dL	94.4 (26.8)	93.5 (24.0)	92.8 (22.0)	92.9 (22.5)	0.027	0.038
Triglyceride, mg/dL	195.0 (99.3)	188.2 (95.4)	183.7 (92.8)	175.1 (89.9)	0.125	0.114
HDL-C, mg/dL	49.7 (11.4)	50.4 (11.6)	50.9 (12.0)	51.5 (12.5)	0.081	0.082
Creatinine, mg/dL	0.8 (0.2)	0.8 (0.2)	0.8 (0.2)	0.8 (0.2)	0.030	0.007
AST, U/L	27.1 (9.8)	27.0 (9.6)	27.0 (10.6)	27.1 (10.6)	0.048	0.007
ALT, U/L	26.9 (14.1)	26.8 (14.6)	26.8 (16.2)	26.7 (16.7)	0.106	0.007
GGT, U/L	42.6 (37.3)	42.3 (39.0)	42.5 (41.6)	42.4 (42.3)	0.030	0.004
Albumin, g/dL	4.5 (0.3)	4.5 (0.3)	4.5 (0.3)	4.5 (0.3)	0.209	0.010
Platelet, $\times 10^9$/L	253.8 (57.4)	254.0 (57.2)	254.2 (58.4)	253.7 (64.3)	0.111	0.004
RDW, %	12.6 (0.3)	13.1 (0.1)	13.6 (0.1)	14.6 (1.0)	2.809	2.834

IPTW-weighted values are weighted mean (SD) for continuous variables and weighted n (%) for categorical variables. IPTW: inverse probability of treatment weighting with stabilized weights from a multinomial propensity model for RDW quartile conditional on age, sex, BMI, diabetes, hypertension, dyslipidemia, current-smoker status, AST, ALT, GGT, platelet, albumin, and creatinine. Weights were trimmed at the 1st and 99th percentiles to reduce the influence of extreme values. IPTW, inverse probability of treatment weighting; RDW, red cell distribution width; BMI, body mass index; HDL-C, high-density lipoprotein cholesterol; AST, aspartate aminotransferase; ALT, alanine aminotransferase; GGT, gamma-glutamyl transferase; SMD, standardized mean difference.

Supplementary Table 10. Baseline characteristics of the MetALD cohort by RDW quartile after IPTW weighting.

Variable	IPTW Q1	IPTW Q2	IPTW Q3	IPTW Q4	SMD (Unweighted)	SMD (IPTW)
Number	10445.4	11323.9	10297.5	7873.9	-	-
Age, years	58.2 (7.5)	58.3 (7.5)	58.3 (7.5)	58.5 (7.4)	0.183	0.015
Male sex	7398.6 (70.8)	8041.2 (71.0)	7313.3 (71.0)	5560.9 (70.6)	0.058	0.005
BMI, kg/m²	28.4 (3.5)	28.4 (3.6)	28.4 (3.6)	28.4 (3.7)	0.087	0.004
Waist circumference, cm	96.5 (9.7)	96.6 (9.7)	96.6 (9.7)	96.9 (9.9)	0.088	0.021
Current smoker	1422.4 (13.6)	1553.9 (13.7)	1414.6 (13.7)	1108.5 (14.1)	0.084	0.007
Diabetes	1034.7 (9.9)	1124.6 (9.9)	1022.5 (9.9)	790.8 (10.0)	0.029	0.002
Hypertension	5360.8 (51.3)	5783.4 (51.1)	5265.0 (51.1)	4070.0 (51.7)	0.056	0.007
Dyslipidemia	3588.4 (34.4)	3907.5 (34.5)	3548.5 (34.5)	2735.6 (34.7)	0.024	0.004
Glucose, mg/dL	94.7 (24.0)	93.5 (20.1)	93.3 (20.3)	92.9 (19.5)	0.034	0.043
Triglyceride, mg/dL	192.6 (100.0)	183.6 (97.0)	176.8 (91.1)	171.1 (92.2)	0.139	0.124
HDL-C, mg/dL	54.4 (12.9)	55.4 (13.1)	56.3 (13.6)	57.4 (14.5)	0.120	0.122
Creatinine, mg/dL	0.9 (0.2)	0.9 (0.2)	0.9 (0.2)	0.9 (0.2)	0.055	0.008
AST, U/L	28.6 (12.7)	28.5 (12.4)	28.5 (13.0)	28.7 (14.0)	0.028	0.008
ALT, U/L	27.9 (15.2)	27.8 (16.1)	27.9 (17.6)	27.6 (17.8)	0.103	0.009
GGT, U/L	55.1 (54.9)	54.7 (57.0)	54.9 (60.3)	55.0 (58.7)	0.035	0.004
Albumin, g/dL	4.5 (0.3)	4.5 (0.3)	4.5 (0.3)	4.5 (0.3)	0.173	0.010
Platelet, ×10⁹/L	247.4 (55.7)	247.7 (56.5)	247.7 (56.0)	247.5 (61.0)	0.080	0.004
RDW, %	12.6 (0.3)	13.1 (0.1)	13.6 (0.1)	14.5 (0.9)	2.897	2.916

IPTW-weighted values are weighted mean (SD) for continuous variables and weighted n (%) for categorical variables. IPTW: inverse probability of treatment weighting with stabilized weights from a multinomial propensity model for RDW quartile conditional on age, sex, BMI, diabetes, hypertension, dyslipidemia, current-smoker status, AST, ALT, GGT, platelet, albumin, and creatinine. Weights were trimmed at the 1st and 99th percentiles to reduce the influence of extreme values. IPTW, inverse probability of treatment weighting; RDW, red cell distribution width; BMI, body mass index; HDL-C, high-density lipoprotein cholesterol; AST, aspartate aminotransferase; ALT, alanine aminotransferase; GGT, gamma-glutamyl transferase; SMD, standardized mean difference.

Supplementary Table 11. IPTW-weighted Cox regression of RDW quartile on clinical outcomes.

Outcome	N events / N	Q1 (ref)	Q2 vs Q1	Q3 vs Q1	Q4 vs Q1	*P*-trend
**Total cohort**						
**Death**	28,483 / 321,247	1.00 (ref)	1.08 (1.04–1.12)<br>*P* < 0.001	1.16 (1.12–1.20)<br>*P* < 0.001	1.42 (1.37–1.47)<br>*P* < 0.001	*P* < 0.001
**Cardiovascular disease**	26,799 / 321,245	1.00 (ref)	1.04 (1.00–1.08)<br>*P* = 0.029	1.08 (1.04–1.12)<br>*P* < 0.001	1.16 (1.12–1.21)<br>*P* < 0.001	*P* < 0.001
**Cerebrovascular disease**	11,880 / 321,247	1.00 (ref)	1.04 (0.99–1.10)<br>*P* = 0.135	1.08 (1.02–1.14)<br>*P* = 0.008	1.19 (1.12–1.25)<br>*P* < 0.001	*P* < 0.001
**Chronic kidney disease**	13,292 / 318,499	1.00 (ref)	1.06 (1.00–1.12)<br>*P* = 0.035	1.09 (1.04–1.15)<br>*P* < 0.001	1.26 (1.20–1.33)<br>*P* < 0.001	*P* < 0.001
**No SLD**						
**Outcome**	**N events / N**	**Q1 (ref)**	**Q2 vs Q1**	**Q3 vs Q1**	**Q4 vs Q1**	***P*-trend**
**Death**	8,207 / 129,605	1.00 (ref)	1.10 (1.03–1.18)<br>*P* = 0.004	1.16 (1.09–1.24)<br>*P* < 0.001	1.39 (1.30–1.48)<br>*P* < 0.001	*P* < 0.001
**Cardiovascular disease**	5,970 / 129,604	1.00 (ref)	1.05 (0.97–1.13)<br>*P* = 0.211	1.04 (0.96–1.12)<br>*P* = 0.339	1.15 (1.07–1.24)<br>*P* < 0.001	*P* < 0.001
**Cerebrovascular disease**	3,444 / 129,605	1.00 (ref)	1.09 (0.99–1.21)<br>*P* = 0.077	1.09 (0.98–1.20)<br>*P* = 0.098	1.17 (1.06–1.29)<br>*P* = 0.002	*P* = 0.004
**Chronic kidney disease**	3,217 / 128,902	1.00 (ref)	1.03 (0.93–1.15)<br>*P* = 0.579	1.08 (0.98–1.20)<br>*P* = 0.131	1.23 (1.11–1.36)<br>*P* < 0.001	*P* < 0.001
**MASLD**						
**Outcome**	**N events / N**	**Q1 (ref)**	**Q2 vs Q1**	**Q3 vs Q1**	**Q4 vs Q1**	***P*-trend**
**Death**	14,944 / 151,560	1.00 (ref)	1.06 (1.01–1.12)<br>*P* = 0.026	1.14 (1.09–1.20)<br>*P* < 0.001	1.40 (1.33–1.48)<br>*P* < 0.001	*P* < 0.001
**Cardiovascular disease**	16,164 / 151,559	1.00 (ref)	1.04 (0.99–1.09)<br>*P* = 0.099	1.10 (1.05–1.15)<br>*P* < 0.001	1.18 (1.12–1.23)<br>*P* < 0.001	*P* < 0.001
**Cerebrovascular disease**	6,349 / 151,560	1.00 (ref)	1.00 (0.93–1.08)<br>*P* = 0.941	1.08 (1.00–1.16)<br>*P* = 0.055	1.18 (1.09–1.27)<br>*P* < 0.001	*P* < 0.001
**Chronic kidney disease**	8,140 / 149,914	1.00 (ref)	1.04 (0.97–1.12)<br>*P* = 0.267	1.05 (0.98–1.12)<br>*P* = 0.187	1.22 (1.14–1.30)<br>*P* < 0.001	*P* < 0.001
**MetALD**						
**Outcome**	**N events / N**	**Q1 (ref)**	**Q2 vs Q1**	**Q3 vs Q1**	**Q4 vs Q1**	***P*-trend**
**Death**	5,332 / 40,082	1.00 (ref)	1.10 (1.02–1.19)<br>*P* = 0.020	1.17 (1.08–1.27)<br>*P* < 0.001	1.45 (1.34–1.58)<br>*P* < 0.001	*P* < 0.001
**Cardiovascular disease**	4,665 / 40,082	1.00 (ref)	1.02 (0.94–1.11)<br>*P* = 0.641	1.09 (1.00–1.18)<br>*P* = 0.048	1.15 (1.05–1.25)<br>*P* = 0.002	*P* < 0.001
**Cerebrovascular disease**	2,087 / 40,082	1.00 (ref)	1.07 (0.94–1.21)<br>*P* = 0.296	1.04 (0.92–1.19)<br>*P* = 0.503	1.21 (1.06–1.38)<br>*P* = 0.004	*P* = 0.012
**Chronic kidney disease**	1,935 / 39,683	1.00 (ref)	1.14 (0.99–1.30)<br>*P* = 0.064	1.22 (1.06–1.39)<br>*P* = 0.005	1.34 (1.17–1.54)<br>*P* < 0.001	*P* < 0.001

Stabilized multinomial propensity weights were computed conditional on age, sex, BMI, diabetes, hypertension, dyslipidemia, current-smoker status, AST, ALT, GGT, platelet, albumin, and creatinine, and trimmed at the 1st and 99th percentiles. HRs and 95% CIs were estimated with robust standard errors. Subjects with previous events at baseline were excluded.

Supplementary Table 12. Mediation and joint Cox marker comparison in the MASLD cohort.

Mediator	Outcome	Events / N	Total HR per 1% RDW (95% CI)	Direct HR (95% CI), with mediator	Proportion mediated (95% CI)	Mediator HR per 1 SD (Joint Cox)
Albumin	Death	15,020 / 152,332	1.16 (1.15–1.18) *P* < 0.001	1.15 (1.14–1.17) *P* < 0.001	5.1% (3.5–7.2)	0.90 (0.88–0.91) *P* < 0.001
	Cardiovascular disease	16,248 / 152,331	1.09 (1.07–1.10) *P* < 0.001	1.08 (1.06–1.09) *P* < 0.001	11.5% (8.8–15.6)	0.91 (0.90–0.93) *P* < 0.001
	Cerebrovascular disease	6,389 / 152,332	1.10 (1.07–1.12) *P* < 0.001	1.08 (1.06–1.11) *P* < 0.001	14.7% (10.3–22.7)	0.87 (0.85–0.90) *P* < 0.001
	Chronic kidney disease	8,188 / 150,679	1.14 (1.12–1.16) *P* < 0.001	1.12 (1.10–1.14) *P* < 0.001	9.0% (6.5–12.4)	0.87 (0.85–0.89) *P* < 0.001
AST	Death	16,240 / 165,124	1.16 (1.15–1.18) *P* < 0.001	1.16 (1.15–1.18) *P* < 0.001	-0.4% (-0.8–-0.2)	1.14 (1.12–1.16) *P* < 0.001
	Cardiovascular disease	17,593 / 165,121	1.09 (1.07–1.10) *P* < 0.001	1.09 (1.07–1.10) *P* < 0.001	0.3% (-0.2–0.9)	1.02 (0.99–1.04) *P* = 0.222
	Cerebrovascular disease	6,938 / 165,124	1.10 (1.07–1.12) *P* < 0.001	1.10 (1.07–1.12) *P* < 0.001	-0.5% (-1.1–0.1)	1.10 (1.06–1.14) *P* < 0.001
	Chronic kidney disease	8,938 / 163,315	1.14 (1.12–1.16) *P* < 0.001	1.14 (1.12–1.16) *P* < 0.001	-0.1% (-0.4–0.2)	1.14 (1.11–1.17) *P* < 0.001
ALT	Death	16,291 / 165,578	1.16 (1.15–1.18) *P* < 0.001	1.16 (1.15–1.17) *P* < 0.001	0.6% (0.1–1.2)	0.83 (0.81–0.86) *P* < 0.001
	Cardiovascular disease	17,645 / 165,575	1.09 (1.07–1.10) *P* < 0.001	1.08 (1.07–1.10) *P* < 0.001	1.9% (0.5–3.7)	0.95 (0.93–0.98) *P* < 0.001
	Cerebrovascular disease	6,956 / 165,578	1.10 (1.07–1.12) *P* < 0.001	1.09 (1.07–1.12) *P* < 0.001	3.3% (1.0–6.7)	0.87 (0.83–0.90) *P* < 0.001
	Chronic kidney disease	8,964 / 163,765	1.14 (1.12–1.16) *P* < 0.001	1.14 (1.12–1.16) *P* < 0.001	3.4% (2.1–4.9)	0.83 (0.80–0.86) *P* < 0.001
GGT	Death	16,299 / 165,700	1.16 (1.15–1.18) *P* < 0.001	1.16 (1.15–1.18) *P* < 0.001	-0.9% (-1.6–-0.2)	1.12 (1.10–1.13) *P* < 0.001
	Cardiovascular disease	17,660 / 165,697	1.09 (1.07–1.10) *P* < 0.001	1.09 (1.07–1.10) *P* < 0.001	-0.5% (-0.9–-0.1)	1.04 (1.03–1.06) *P* < 0.001
	Cerebrovascular disease	6,961 / 165,700	1.10 (1.07–1.12) *P* < 0.001	1.10 (1.07–1.12) *P* < 0.001	-0.8% (-1.5–-0.2)	1.07 (1.05–1.10) *P* < 0.001
	Chronic kidney disease	8,972 / 163,887	1.14 (1.12–1.16) *P* < 0.001	1.14 (1.12–1.16) *P* < 0.001	-0.4% (-0.8–-0.1)	1.07 (1.05–1.09) *P* < 0.001
Total bilirubin	Death	16,241 / 165,145	1.16 (1.15–1.18) *P* < 0.001	1.16 (1.15–1.17) *P* < 0.001	0.4% (-0.0–0.9)	0.97 (0.95–0.99) *P* = 0.001
	Cardiovascular disease	17,599 / 165,142	1.09 (1.07–1.10) *P* < 0.001	1.09 (1.07–1.10) *P* < 0.001	0.7% (-0.2–1.6)	1.01 (0.99–1.02) *P* = 0.379
	Cerebrovascular disease	6,939 / 165,145	1.10 (1.07–1.12) *P* < 0.001	1.10 (1.07–1.12) *P* < 0.001	0.7% (-0.3–2.1)	0.99 (0.97–1.02) *P* = 0.697
	Chronic kidney disease	8,941 / 163,336	1.14 (1.12–1.16) *P* < 0.001	1.14 (1.12–1.16) *P* < 0.001	0.3% (-0.2–0.9)	1.01 (0.98–1.03) *P* = 0.531
Platelet	Death	16,299 / 165,699	1.16 (1.15–1.18) *P* < 0.001	1.16 (1.14–1.17) *P* < 0.001	2.8% (1.7–4.1)	1.05 (1.03–1.07) *P* < 0.001
	Cardiovascular disease	17,660 / 165,696	1.09 (1.07–1.10) *P* < 0.001	1.08 (1.07–1.10) *P* < 0.001	3.2% (1.2–5.4)	1.02 (1.01–1.04) *P* = 0.003
	Cerebrovascular disease	6,961 / 165,699	1.10 (1.07–1.12) *P* < 0.001	1.09 (1.07–1.12) *P* < 0.001	4.4% (1.4–8.3)	1.04 (1.01–1.07) *P* = 0.003
	Chronic kidney disease	8,972 / 163,886	1.14 (1.12–1.16) *P* < 0.001	1.14 (1.13–1.16) *P* < 0.001	-2.0% (-3.9–-0.5)	1.01 (0.99–1.03) *P* = 0.417
Glucose	Death	15,014 / 152,204	1.16 (1.15–1.18) *P* < 0.001	1.16 (1.15–1.18) *P* < 0.001	-1.2% (-1.8–-0.7)	1.06 (1.05–1.08) *P* < 0.001
	Cardiovascular disease	16,237 / 152,203	1.09 (1.07–1.10) *P* < 0.001	1.09 (1.07–1.10) *P* < 0.001	0.5% (-0.2–1.1)	0.98 (0.97–1.00) *P* = 0.028
	Cerebrovascular disease	6,381 / 152,204	1.10 (1.07–1.12) *P* < 0.001	1.10 (1.07–1.12) *P* < 0.001	-0.6% (-1.6–0.3)	1.01 (0.99–1.04) *P* = 0.186

Mediator	Outcome	Events / N	Total HR per 1% RDW (95% CI)	Direct HR (95% CI), with mediator	Proportion mediated (95% CI)	Mediator HR per 1 SD (Joint Cox)
	Chronic kidney disease	8,178 / 150,552	1.14 (1.12–1.16) *P* < 0.001	1.14 (1.12–1.16) *P* < 0.001	-1.4% (-2.3–-0.7)	1.07 (1.05–1.08) *P* < 0.001
Triglyceride	Death	16,299 / 165,700	1.16 (1.15–1.18) *P* < 0.001	1.16 (1.15–1.18) *P* < 0.001	-0.2% (-0.8–0.4)	0.99 (0.97–1.01) *P* = 0.339
	Cardiovascular disease	17,660 / 165,697	1.09 (1.07–1.10) *P* < 0.001	1.09 (1.08–1.11) *P* < 0.001	-5.7% (-7.8–-3.9)	1.05 (1.03–1.07) *P* < 0.001
	Cerebrovascular disease	6,961 / 165,700	1.10 (1.07–1.12) *P* < 0.001	1.10 (1.07–1.12) *P* < 0.001	1.3% (-0.7–3.5)	0.99 (0.96–1.01) *P* = 0.321
	Chronic kidney disease	8,972 / 163,887	1.14 (1.12–1.16) *P* < 0.001	1.15 (1.13–1.16) *P* < 0.001	-2.3% (-3.6–-1.3)	1.04 (1.01–1.06) *P* = 0.003
HDL-C	Death	15,027 / 152,295	1.16 (1.15–1.18) *P* < 0.001	1.16 (1.15–1.18) *P* < 0.001	-0.3% (-0.6–0.0)	0.96 (0.94–0.97) *P* < 0.001
	Cardiovascular disease	16,247 / 152,294	1.09 (1.07–1.10) *P* < 0.001	1.09 (1.07–1.10) *P* < 0.001	-2.7% (-4.0–-1.6)	0.94 (0.92–0.95) *P* < 0.001
	Cerebrovascular disease	6,388 / 152,295	1.10 (1.07–1.12) *P* < 0.001	1.10 (1.07–1.12) *P* < 0.001	-0.5% (-1.5–0.2)	0.98 (0.95–1.01) *P* = 0.165
	Chronic kidney disease	8,189 / 150,642	1.14 (1.12–1.16) *P* < 0.001	1.14 (1.12–1.16) *P* < 0.001	-1.1% (-2.1–-0.2)	0.94 (0.91–0.96) *P* < 0.001
Creatinine	Death	16,295 / 165,681	1.16 (1.15–1.18) *P* < 0.001	1.16 (1.15–1.17) *P* < 0.001	1.5% (0.8–2.3)	1.04 (1.04–1.05) *P* < 0.001
	Cardiovascular disease	17,659 / 165,678	1.09 (1.07–1.10) *P* < 0.001	1.08 (1.07–1.10) *P* < 0.001	4.1% (2.4–6.1)	1.04 (1.03–1.05) *P* < 0.001
	Cerebrovascular disease	6,958 / 165,681	1.10 (1.07–1.12) *P* < 0.001	1.09 (1.07–1.12) *P* < 0.001	3.0% (1.4–5.3)	1.04 (1.02–1.05) *P* < 0.001
	Chronic kidney disease	8,969 / 163,868	1.14 (1.12–1.16) *P* < 0.001	1.12 (1.10–1.14) *P* < 0.001	17.5% (9.0–57.6)	1.38 (1.37–1.39) *P* < 0.001

Combined summary of mediation analysis and joint Cox marker comparison in the MASLD cohort. Total HR is the unadjusted (Model 3-confounder-adjusted) Cox hazard ratio for RDW per 1% increase. Direct HR is the same exposure effect after additionally adjusting for the candidate mediator. PM (proportion mediated) = (log Total HR − log Direct HR) / log Total HR × 100; bootstrap 95% CI (B = 500). Mediator's own HR per 1 SD comes from the joint Cox model containing RDW + all 10 mediators + Model 3 covariates simultaneously. All models adjusted for age, sex, BMI, diabetes, hypertension, dyslipidemia, and smoking status. RDW, red cell distribution width; HR, hazard ratio; CI, confidence interval; PM, proportion mediated; SD, standard deviation; AST, aspartate aminotransferase; ALT, alanine aminotransferase; GGT, gamma-glutamyl transferase; HDL-C, high-density lipoprotein cholesterol; MASLD, metabolic dysfunction–associated steatotic liver disease.

Supplementary Table 13. Cox regression of RDW per 1% across clinical outcomes, stratified by FIB-4 category in the MASLD cohort.

Outcome	Form	Model 1	Model 2	Model 3	Model 4
**Low**	Death	1.18 (1.17–1.20)<br>*P* < 0.001	1.17 (1.15–1.18)<br>*P* < 0.001	1.15 (1.13–1.17)<br>*P* < 0.001	1.12 (1.10–1.14)<br>*P* < 0.001
	Cardiovascular disease	1.11 (1.10–1.13)<br>*P* < 0.001	1.12 (1.10–1.13)<br>*P* < 0.001	1.10 (1.07–1.12)<br>*P* < 0.001	1.07 (1.05–1.10)<br>*P* < 0.001
	Cerebrovascular disease	1.15 (1.12–1.17)<br>*P* < 0.001	1.12 (1.09–1.15)<br>*P* < 0.001	1.11 (1.07–1.14)<br>*P* < 0.001	1.07 (1.03–1.11)<br>*P* < 0.001
	Chronic kidney disease	1.19 (1.17–1.21)<br>*P* < 0.001	1.16 (1.14–1.18)<br>*P* < 0.001	1.13 (1.11–1.16)<br>*P* < 0.001	1.08 (1.05–1.11)<br>*P* < 0.001
**Indeterminate**	Death	1.21 (1.19–1.23)<br>*P* < 0.001	1.22 (1.19–1.24)<br>*P* < 0.001	1.17 (1.15–1.20)<br>*P* < 0.001	1.15 (1.12–1.17)<br>*P* < 0.001
	Cardiovascular disease	1.12 (1.09–1.14)<br>*P* < 0.001	1.11 (1.08–1.13)<br>*P* < 0.001	1.07 (1.05–1.10)<br>*P* < 0.001	1.05 (1.03–1.08)<br>*P* < 0.001
	Cerebrovascular disease	1.15 (1.11–1.18)<br>*P* < 0.001	1.12 (1.08–1.16)<br>*P* < 0.001	1.08 (1.05–1.13)<br>*P* < 0.001	1.05 (1.01–1.10)<br>*P* = 0.010
	Chronic kidney disease	1.21 (1.18–1.23)<br>*P* < 0.001	1.21 (1.18–1.24)<br>*P* < 0.001	1.16 (1.13–1.19)<br>*P* < 0.001	1.06 (1.02–1.09)<br>*P* = 0.002
**High**	Death	1.21 (1.16–1.26)<br>*P* < 0.001	1.22 (1.17–1.28)<br>*P* < 0.001	1.21 (1.16–1.26)<br>*P* < 0.001	1.20 (1.15–1.26)<br>*P* < 0.001
	Cardiovascular disease	1.08 (1.01–1.15)<br>*P* = 0.025	1.09 (1.02–1.16)<br>*P* = 0.014	1.07 (1.00–1.15)<br>*P* = 0.041	1.07 (0.99–1.15)<br>*P* = 0.072
	Cerebrovascular disease	1.09 (1.00–1.20)<br>*P* = 0.053	1.10 (1.00–1.21)<br>*P* = 0.045	1.09 (0.99–1.20)<br>*P* = 0.072	1.09 (0.98–1.21)<br>*P* = 0.123
	Chronic kidney disease	1.12 (1.04–1.21)<br>*P* = 0.002	1.13 (1.05–1.22)<br>*P* = 0.002	1.10 (1.02–1.19)<br>*P* = 0.011	1.06 (0.96–1.17)<br>*P* = 0.274

Hazard ratios (HR) per 1% increase in RDW from Cox proportional hazards models, stratified by FIB-4 category in the MASLD cohort. FIB-4 categories: Low (<1.30), Indeterminate (1.30–2.67), High (≥2.67). Model 1: crude. Model 2: adjusted for age and sex. Model 3: Model 2 + BMI, diabetes, hypertension, dyslipidemia, and smoking status. Model 4: Model 3 + AST, ALT, GGT, platelet count, albumin, and creatinine. Previous events at baseline were excluded from each outcome analysis. RDW, red cell distribution width; FIB-4, fibrosis-4 index; HR, hazard ratio; CI, confidence interval; BMI, body mass index; AST, aspartate aminotransferase; ALT, alanine aminotransferase; GGT, gamma-glutamyl transferase; MASLD, metabolic dysfunction–associated steatotic liver disease.

Supplementary Table 14. Cox regression of RDW per 1% across clinical outcomes, stratified by NFS category in the MASLD cohort.

Outcome	Form	Model 1	Model 2	Model 3	Model 4
**Low**	Death	1.18 (1.16–1.19)<br>*P* < 0.001	1.17 (1.15–1.18)<br>*P* < 0.001	1.15 (1.13–1.17)<br>*P* < 0.001	1.12 (1.10–1.14)<br>*P* < 0.001
	Cardiovascular disease	1.09 (1.07–1.11)<br>*P* < 0.001	1.09 (1.07–1.12)<br>*P* < 0.001	1.08 (1.06–1.11)<br>*P* < 0.001	1.06 (1.04–1.09)<br>*P* < 0.001
	Cerebrovascular disease	1.13 (1.10–1.16)<br>*P* < 0.001	1.11 (1.08–1.15)<br>*P* < 0.001	1.10 (1.06–1.14)<br>*P* < 0.001	1.06 (1.02–1.10)<br>*P* < 0.001
	Chronic kidney disease	1.17 (1.15–1.20)<br>*P* < 0.001	1.15 (1.12–1.17)<br>*P* < 0.001	1.14 (1.12–1.17)<br>*P* < 0.001	1.05 (1.01–1.08)<br>*P* = 0.010
**Indeterminate**	Death	1.17 (1.15–1.19)<br>*P* < 0.001	1.20 (1.18–1.22)<br>*P* < 0.001	1.17 (1.14–1.19)<br>*P* < 0.001	1.14 (1.12–1.17)<br>*P* < 0.001
	Cardiovascular disease	1.09 (1.07–1.12)<br>*P* < 0.001	1.11 (1.09–1.14)<br>*P* < 0.001	1.09 (1.06–1.11)<br>*P* < 0.001	1.07 (1.05–1.10)<br>*P* < 0.001
	Cerebrovascular disease	1.10 (1.06–1.14)<br>*P* < 0.001	1.11 (1.07–1.15)<br>*P* < 0.001	1.08 (1.05–1.12)<br>*P* < 0.001	1.06 (1.02–1.10)<br>*P* = 0.002
	Chronic kidney disease	1.16 (1.14–1.19)<br>*P* < 0.001	1.17 (1.14–1.20)<br>*P* < 0.001	1.13 (1.10–1.16)<br>*P* < 0.001	1.09 (1.06–1.12)<br>*P* < 0.001
**High**	Death	1.17 (1.12–1.22)<br>*P* < 0.001	1.19 (1.14–1.24)<br>*P* < 0.001	1.19 (1.13–1.24)<br>*P* < 0.001	1.16 (1.10–1.22)<br>*P* < 0.001
	Cardiovascular disease	1.09 (1.02–1.15)<br>*P* = 0.007	1.11 (1.05–1.17)<br>*P* < 0.001	1.09 (1.02–1.16)<br>*P* = 0.007	1.06 (0.99–1.13)<br>*P* = 0.103
	Cerebrovascular disease	1.13 (1.04–1.22)<br>*P* = 0.003	1.16 (1.07–1.25)<br>*P* < 0.001	1.15 (1.06–1.24)<br>*P* < 0.001	1.13 (1.04–1.23)<br>*P* = 0.006
	Chronic kidney disease	1.12 (1.05–1.19)<br>*P* < 0.001	1.14 (1.07–1.21)<br>*P* < 0.001	1.12 (1.05–1.20)<br>*P* < 0.001	1.08 (0.99–1.17)<br>*P* = 0.071

Hazard ratios (HR) per 1% increase in RDW from Cox proportional hazards models, stratified by FIB-4 category in the MASLD cohort. FIB-4 categories: Low (<1.30), Indeterminate (1.30–2.67), High (≥2.67). Model 1: crude. Model 2: adjusted for age and sex. Model 3: Model 2 + BMI, diabetes, hypertension, dyslipidemia, and smoking status. Model 4: Model 3 + AST, ALT, GGT, platelet count, albumin, and creatinine. Previous events at baseline were excluded from each outcome analysis. RDW, red cell distribution width; NFS, NAFLD fibrosis score; HR, hazard ratio; CI, confidence interval; BMI, body mass index; AST, aspartate aminotransferase; ALT, alanine aminotransferase; GGT, gamma-glutamyl transferase; MASLD, metabolic dysfunction–associated steatotic liver disease.
